# Supplementary material for: Centromere evolution and CpG methylation during vertebrate speciation
Source: Nat Commun. 2017 Nov 28;8:1833. doi: 10.1038/s41467-017-01982-7 (PMC5705604; doi:10.1038/s41467-017-01982-7)
Supplement: Supplementary file 1 — Supplementary Information [file 41467_2017_1982_MOESM1_ESM.pdf]

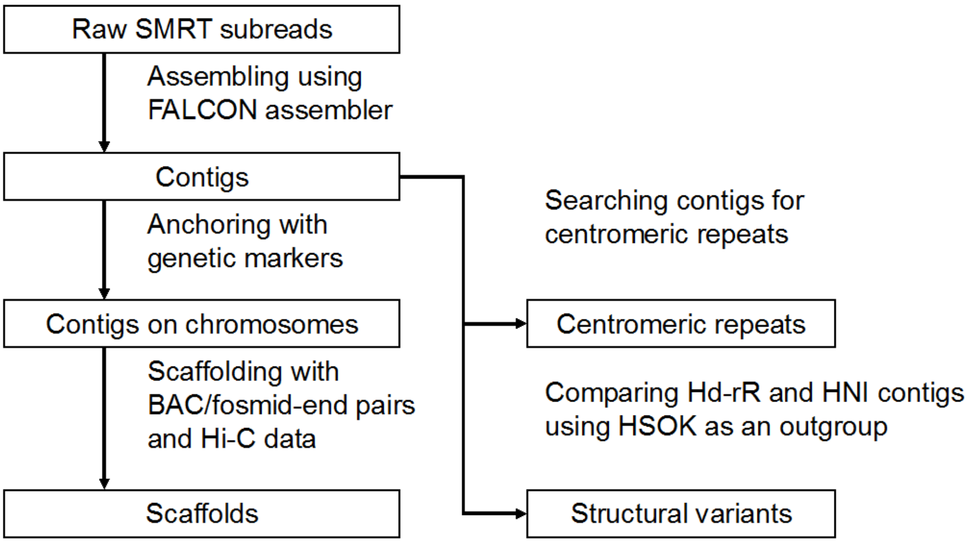

Supplementary Figure 1: Information processing pipeline

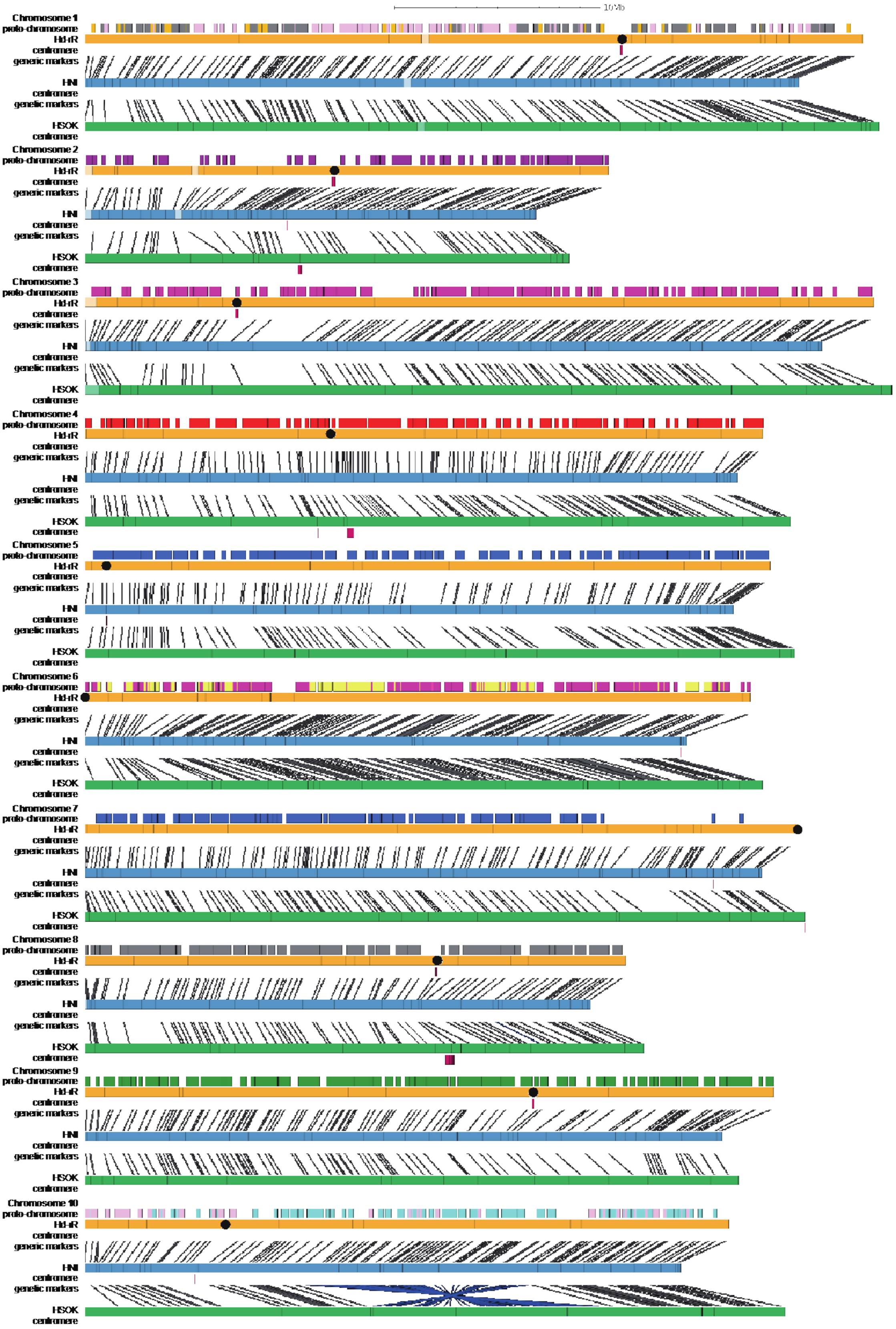
10 Mb
Chromosome 1
proto-chromosome
HdrR
centromere
genetic markers
HNI
centromere
genetic markers
HSOK
centromere
Chromosome 2
proto-chromosome
HdrR
centromere
genetic markers
HNI
centromere
genetic markers
HSOK
centromere
Chromosome 3
proto-chromosome
HdrR
centromere
genetic markers
HNI
centromere
genetic markers
HSOK
centromere
Chromosome 4
proto-chromosome
HdrR
centromere
genetic markers
HNI
centromere
genetic markers
HSOK
centromere
Chromosome 5
proto-chromosome
HdrR
centromere
genetic markers
HNI
centromere
genetic markers
HSOK
centromere
Chromosome 6
proto-chromosome
HdrR
centromere
genetic markers
HNI
centromere
genetic markers
HSOK
centromere
Chromosome 7
proto-chromosome
HdrR
centromere
genetic markers
HNI
centromere
genetic markers
HSOK
centromere
Chromosome 8
proto-chromosome
HdrR
centromere
genetic markers
HNI
centromere
genetic markers
HSOK
centromere
Chromosome 9
proto-chromosome
HdrR
centromere
genetic markers
HNI
centromere
genetic markers
HSOK
centromere
Chromosome 10
proto-chromosome
HdrR
centromere
genetic markers
HNI
centromere
genetic markers
HSOK
centromere

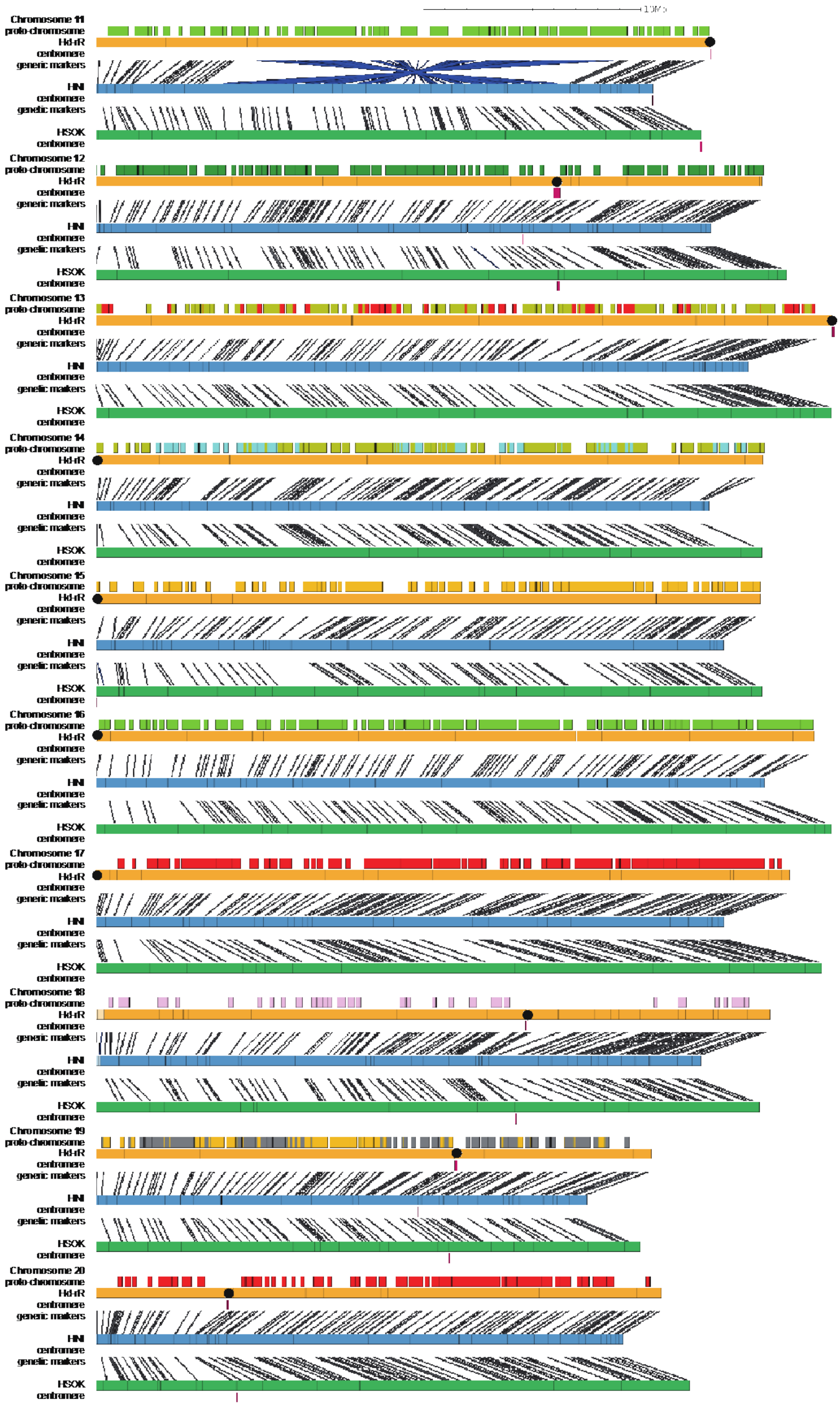

10Mb
Chromosome 11
proto-chromosome
Hd-rR
centromere
genetic markers
HNI
centromere
genetic markers
HSOK
centromere
Chromosome 12
proto-chromosome
Hd-rR
centromere
genetic markers
HNI
centromere
genetic markers
HSOK
centromere
Chromosome 13
proto-chromosome
Hd-rR
centromere
genetic markers
HNI
centromere
genetic markers
HSOK
centromere
Chromosome 14
proto-chromosome
Hd-rR
centromere
genetic markers
HNI
centromere
genetic markers
HSOK
centromere
Chromosome 15
proto-chromosome
Hd-rR
centromere
genetic markers
HNI
centromere
genetic markers
HSOK
centromere
Chromosome 16
proto-chromosome
Hd-rR
centromere
genetic markers
HNI
centromere
genetic markers
HSOK
centromere
Chromosome 17
proto-chromosome
Hd-rR
centromere
genetic markers
HNI
centromere
genetic markers
HSOK
centromere
Chromosome 18
proto-chromosome
Hd-rR
centromere
genetic markers
HNI
centromere
genetic markers
HSOK
centromere
Chromosome 19
proto-chromosome
Hd-rR
centromere
genetic markers
HNI
centromere
genetic markers
HSOK
centromere
Chromosome 20
proto-chromosome
Hd-rR
centromere
genetic markers
HNI
centromere
genetic markers
HSOK
centromere

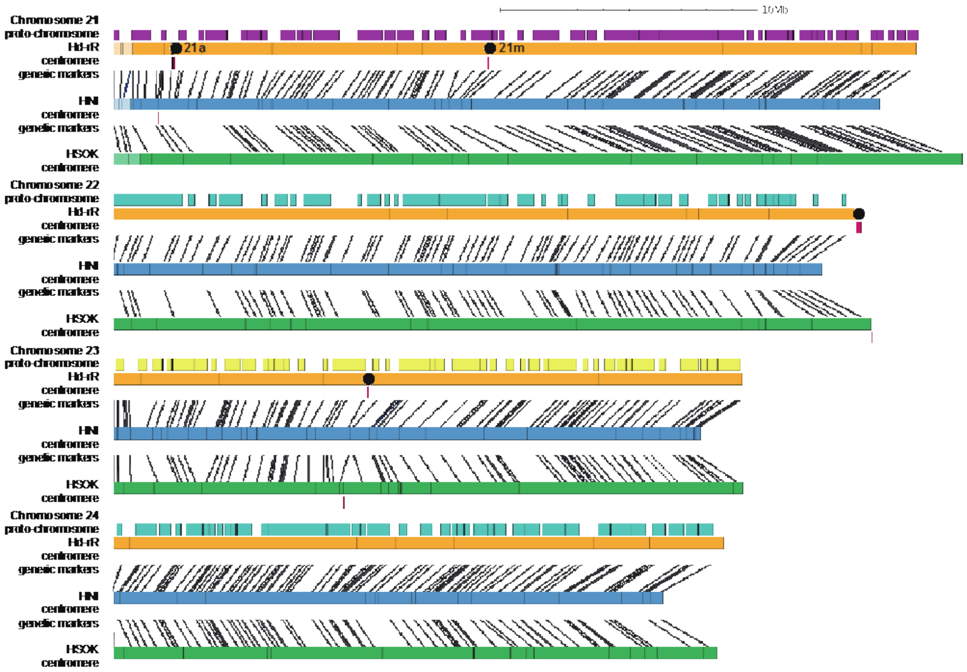

Supplementary Figure 2: Assembled contigs and scaffolds for medaka chromosomes of Hd-rR, HNI and HSOK. The orange boxes are scaffolds for Hd-rR, and the other blue and green boxes are contigs of HNI and HSOK. Most of contigs are oriented, but light-colored boxes remain non-oriented in chromosomes because they have only single markers, or sets of genetic markers at the same genetic distance. Pink bars below contigs display centromeric repeats identified (see general statistics in Supplementary Table 12, and positions in Supplementary Table 13). The gray lines connecting contig boxes reflect the correspondence between genetic markers anchored on contigs. In chr. 2, 8, 11, 12, 18-20, 22, and 23 in which centromeric repeats were sequenced, centromeric repeats were located at identical genetic loci between multiple strains, and these genetic loci are denoted by black solid circles on Hd-rR orange boxes. In chr. 1, 3, 5, 9, 10, 13-17, only a single strain had a centromeric repeat region, and we put a black circle on the generic locus in the Hd-rR chromosome. Chr. 4, 6, 7, 21, and 24 are exceptional. Chromosome 4 had one ~10Kbp region at a Hd-rR acrocentric position and another ~305Kbp region at a HSOK metacentric region, and the latter longer region is labeled with a black circle. Chromosome 6 is an acrocentric chromosome, and intriguingly Hd-rR and HSOK have centromeric repeats at the same genetic loci, while HNI has repeats at the opposite end, and we therefore put a black circle at the acrocentric position in Hd-rR. Chromosome 7 of both HNI and HSOK is acrocentric, but those regions are not at an identical generic locus. Chromosome 21 had two centromeric repeat regions at Hd-rR acrocentric and metacentric positions, and respective loci are denoted by 21a and 21m. No centromeric repeats were found in chromosome 24 in any of the three strains.

a

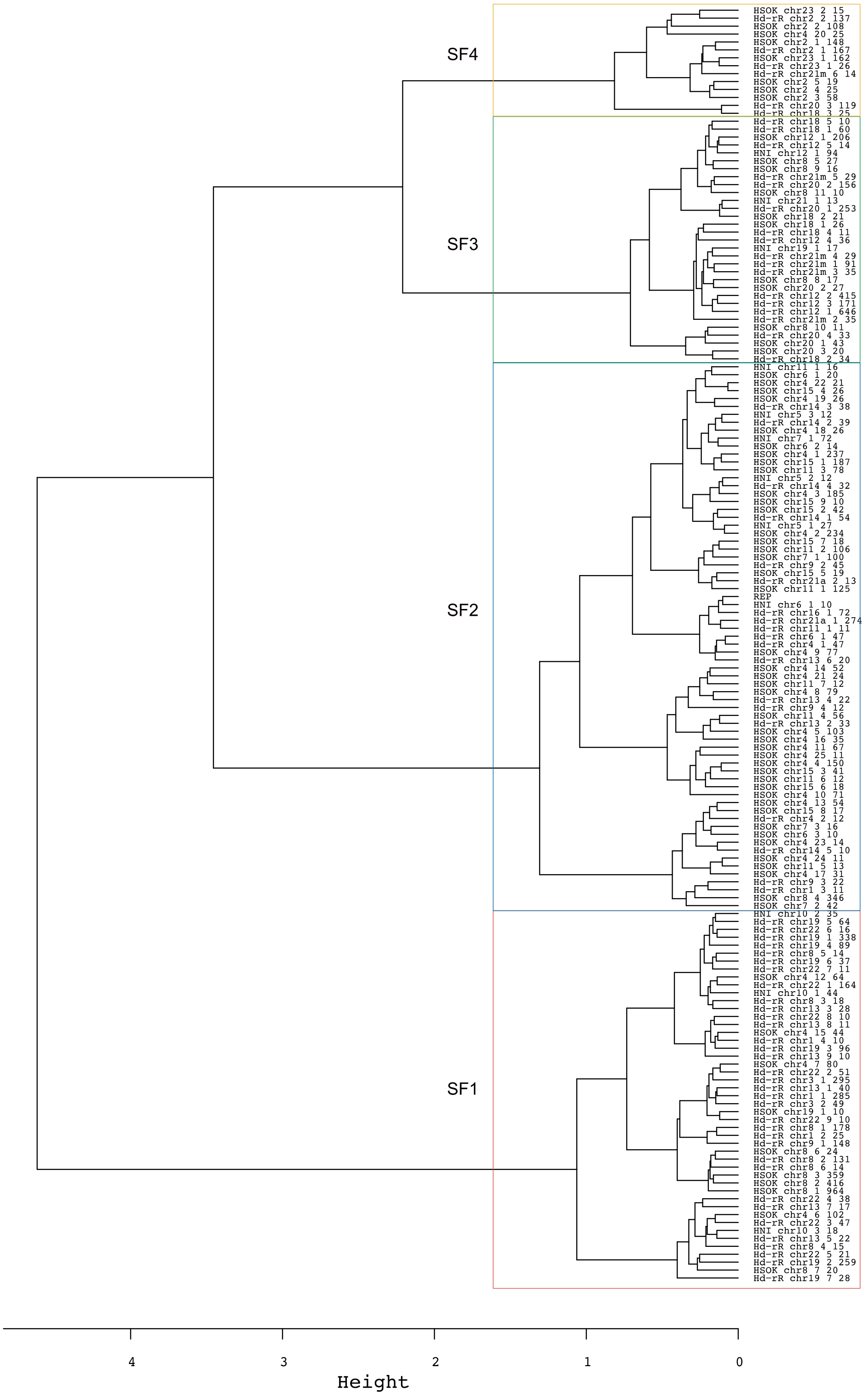

SF4
SF3
SF2
SF1
HSOK chr23 2 15
Hd-rR chr2 2 137
HSOK chr2 2 108
HSOK chr4 20 25
HSOK chr2 1 148
Hd-rR chr2 1 167
HSOK chr23 1 162
Hd-rR chr23 1 26
Hd-rR chr21m 6 14
HSOK chr2 5 19
HSOK chr2 4 25
HSOK chr2 3 58
Hd-rR chr20 3 119
Hd-rR chr18 3 25
Hd-rR chr18 5 10
Hd-rR chr18 1 60
HSOK chr12 1 206
Hd-rR chr12 5 14
HNI chr12 1 94
HSOK chr8 5 27
HSOK chr8 9 16
Hd-rR chr21m 5 29
Hd-rR chr20 2 156
HSOK chr8 11 10
HNI chr21 1 13
Hd-rR chr20 1 253
HSOK chr18 2 21
HSOK chr18 1 26
Hd-rR chr18 4 11
Hd-rR chr12 4 36
HNI chr19 1 17
Hd-rR chr21m 4 29
Hd-rR chr21m 1 91
Hd-rR chr21m 3 35
HSOK chr8 8 17
HSOK chr20 2 27
Hd-rR chr12 2 415
Hd-rR chr12 3 171
Hd-rR chr12 1 646
Hd-rR chr21m 2 35
HSOK chr8 10 11
Hd-rR chr20 4 33
HSOK chr20 1 43
HSOK chr20 3 20
Hd-rR chr18 2 34
HNI chr11 1 16
HSOK chr6 1 20
HSOK chr4 22 21
HSOK chr15 4 26
HSOK chr4 19 26
Hd-rR chr14 3 38
HNI chr5 3 12
Hd-rR chr14 2 39
HSOK chr4 18 26
HNI chr7 1 72
HSOK chr6 2 14
HSOK chr4 1 237
HSOK chr15 1 187
HSOK chr11 3 78
HNI chr5 2 12
Hd-rR chr14 4 32
HSOK chr4 3 185
HSOK chr15 9 10
HSOK chr15 2 42
Hd-rR chr14 1 54
HNI chr5 1 27
HSOK chr4 2 234
HSOK chr15 7 18
HSOK chr11 2 106
HSOK chr7 1 100
Hd-rR chr9 2 45
HSOK chr15 5 19
Hd-rR chr21a 2 13
HSOK chr11 1 125
REP
HNI chr6 1 10
Hd-rR chr16 1 72
Hd-rR chr21a 1 274
Hd-rR chr11 1 11
Hd-rR chr6 1 47
Hd-rR chr4 1 47
HSOK chr4 9 77
Hd-rR chr13 6 20
HSOK chr4 14 52
HSOK chr4 21 24
HSOK chr11 7 12
HSOK chr4 8 79
Hd-rR chr13 4 22
Hd-rR chr9 4 12
HSOK chr11 4 56
Hd-rR chr13 2 33
HSOK chr4 5 103
HSOK chr4 16 35
HSOK chr4 11 67
HSOK chr4 25 11
HSOK chr4 4 150
HSOK chr15 3 41
HSOK chr11 6 12
HSOK chr15 6 18
HSOK chr4 10 71
HSOK chr4 13 54
HSOK chr15 8 17
Hd-rR chr4 2 12
HSOK chr7 3 16
HSOK chr6 3 10
HSOK chr4 23 14
Hd-rR chr14 5 10
HSOK chr4 24 11
HSOK chr11 5 13
HSOK chr4 17 31
Hd-rR chr9 3 22
Hd-rR chr1 3 11
HSOK chr8 4 346
HSOK chr7 2 42
HNI chr10 2 35
Hd-rR chr19 5 64
Hd-rR chr22 6 16
Hd-rR chr19 1 338
Hd-rR chr19 4 89
Hd-rR chr8 5 14
Hd-rR chr19 6 37
Hd-rR chr22 7 11
HSOK chr4 12 64
Hd-rR chr22 1 164
HNI chr10 1 44
Hd-rR chr8 3 18
Hd-rR chr13 3 28
Hd-rR chr22 8 10
Hd-rR chr13 8 11
HSOK chr4 15 44
Hd-rR chr1 4 10
Hd-rR chr19 3 96
Hd-rR chr13 9 10
HSOK chr4 7 80
Hd-rR chr22 2 51
Hd-rR chr3 1 295
Hd-rR chr13 1 40
Hd-rR chr1 1 285
Hd-rR chr3 2 49
HSOK chr19 1 10
Hd-rR chr22 9 10
Hd-rR chr8 1 178
Hd-rR chr1 2 25
Hd-rR chr9 1 148
HSOK chr8 6 24
Hd-rR chr8 2 131
Hd-rR chr8 6 14
HSOK chr8 3 359
HSOK chr8 2 416
HSOK chr8 1 964
Hd-rR chr22 4 38
Hd-rR chr13 7 17
HSOK chr4 6 102
Hd-rR chr22 3 47
HNI chr10 3 18
Hd-rR chr13 5 22
Hd-rR chr8 4 15
Hd-rR chr22 5 21
Hd-rR chr19 2 259
HSOK chr8 7 20
Hd-rR chr19 7 28
4
3
2
1
0
Height

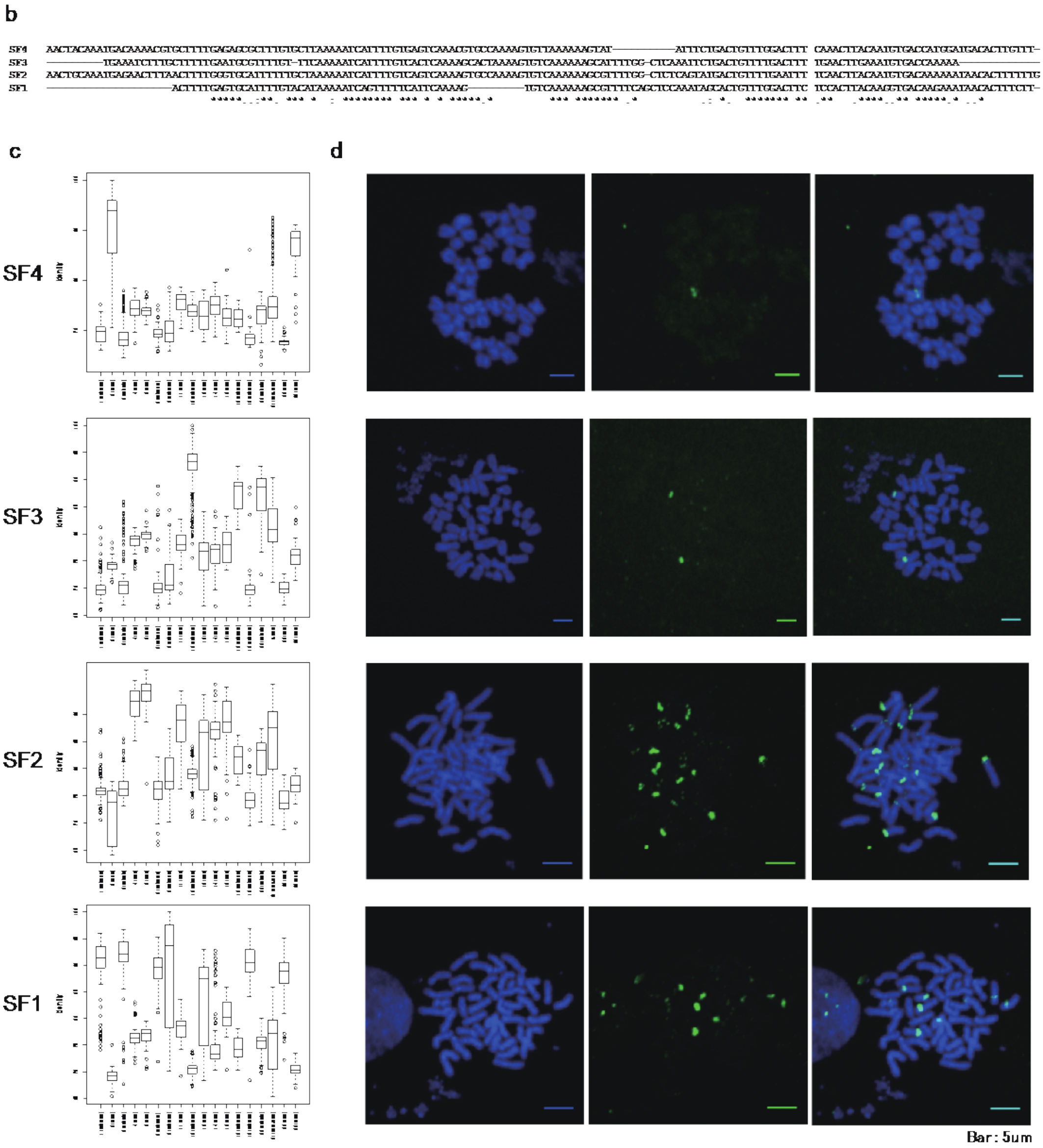
b
SF4
SF3
SF2
SF1
c
d
SF4
SF3
SF2
SF1
Bar: 5um

Supplementary Figure 3: **Diversity of centromeric monomers in Hd-rR chromosomes: a.** A hierarchical clustering of monomers in all chromosomes of the three strains by using DNACLUST. Each leaf has a cluster with $\geq$10 monomers; for example, the top leaf labeled with "HSOK chr23 2 15" indicates the 2nd largest cluster among all clusters in HSOK chromosome 23 with 15 monomers,. The second top leaf with "Hd-rR chr2 2 137" has 137 monomers and is the 2nd largest cluster in Hd-rR chromosome 2. We partitioned all clusters into four groups named SF1-4 enclosed in boxes according to the hierarchical clustering, where SF denotes Supra-chromosomal Family. **b**. From each of the four groups, we selected a representative (Methods). To highlight the difference, we generated a multiple sequence alignment of the representatives using CLUSTAL 2.1. **c.** The four boxplots illustrate the distribution of sequence identity between each representative of SF1-4 and all monomers in individual chromosomes that had centromeric repeats in its genomic sequence (Supplementary Table 12). The x-axis shows chromosome numbers with approximate genomic positions (metacentric, submetacentric, acrocentric, or subtelocentric) of their centromeres in parentheses, and the y-axis displays sequence identity (in percentage). **d**. Fluorescence in situ hybridization (FISH) images. We used the four representative sequences in Figure b as centromere-specific DNA probes. DNA was strained blue with DAPI (left), probes green (middle), and two images were overlaid (right). For example, the representative probe for SF2 matches acrocentric chromosomes with high identities (Figure c), and indeed, green probes are observed at acrocentric positions. Similarly, the respective probes for SF3 and SF4 are seen at submetacentric and metacentric positions. The probe for SF1 that matches submetacentric and acrocentric chromosomes, and hence green probes are found at both submetacentric and acrocentric positions.

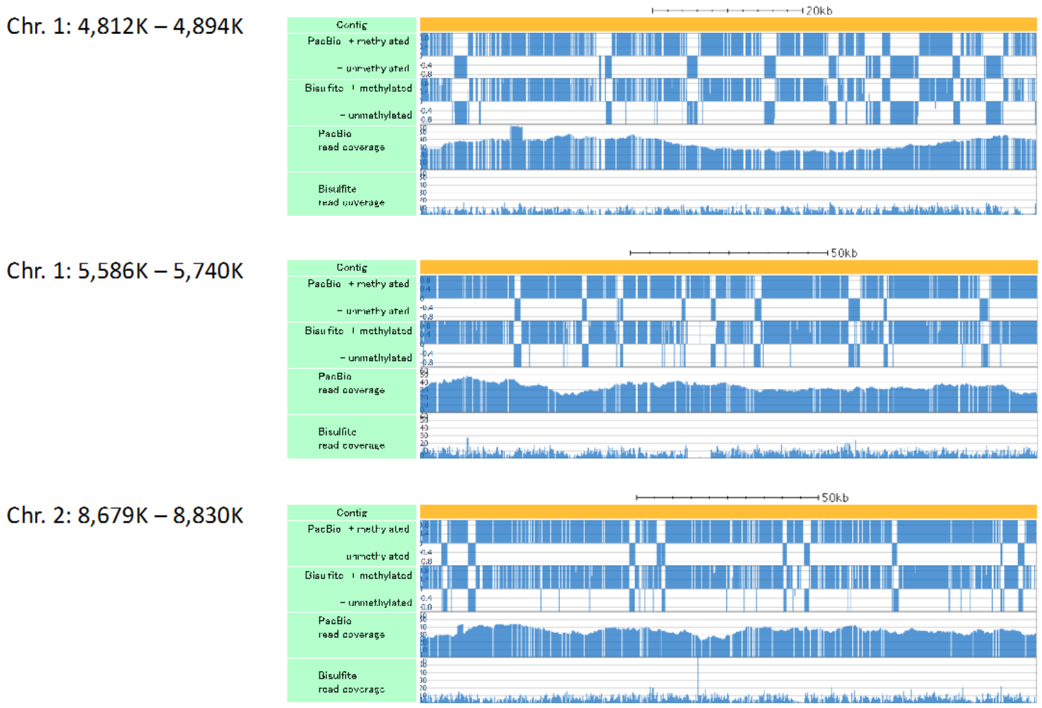

Supplementary Figure 4: **Three examples of genomic regions where CpG methylation states by PacBio sequencing and bisulfite sequencing are almost consistent.** We display tracks for regional methylation prediction from PacBio reads (+, methylated; -, unmethylated), CpG-wise methylation from bisulfite reads, coverage of PacBio reads, and coverage of bisulfite reads in the Hd-rR genome.

Hd-rR chr.1

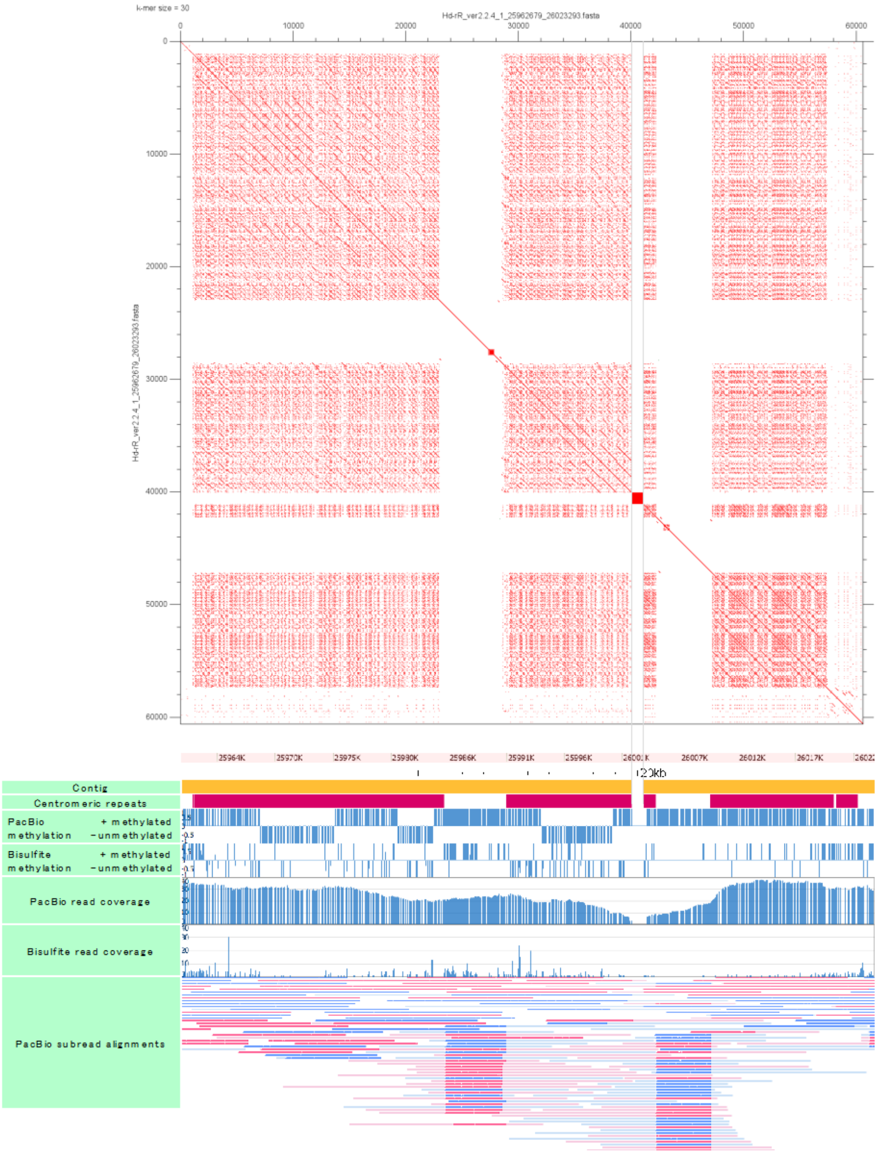

k-mer size = 30
Hd-rR_ver2.2.4_1_25962679_26023293.fasta
Contig
Centromeric repeats
PacBio methylation + methylated - unmethylated
Bisulfite methylation + methylated - unmethylated
PacBio read coverage
Bisulfite read coverage
PacBio subread alignments

Hd-rR chr.2

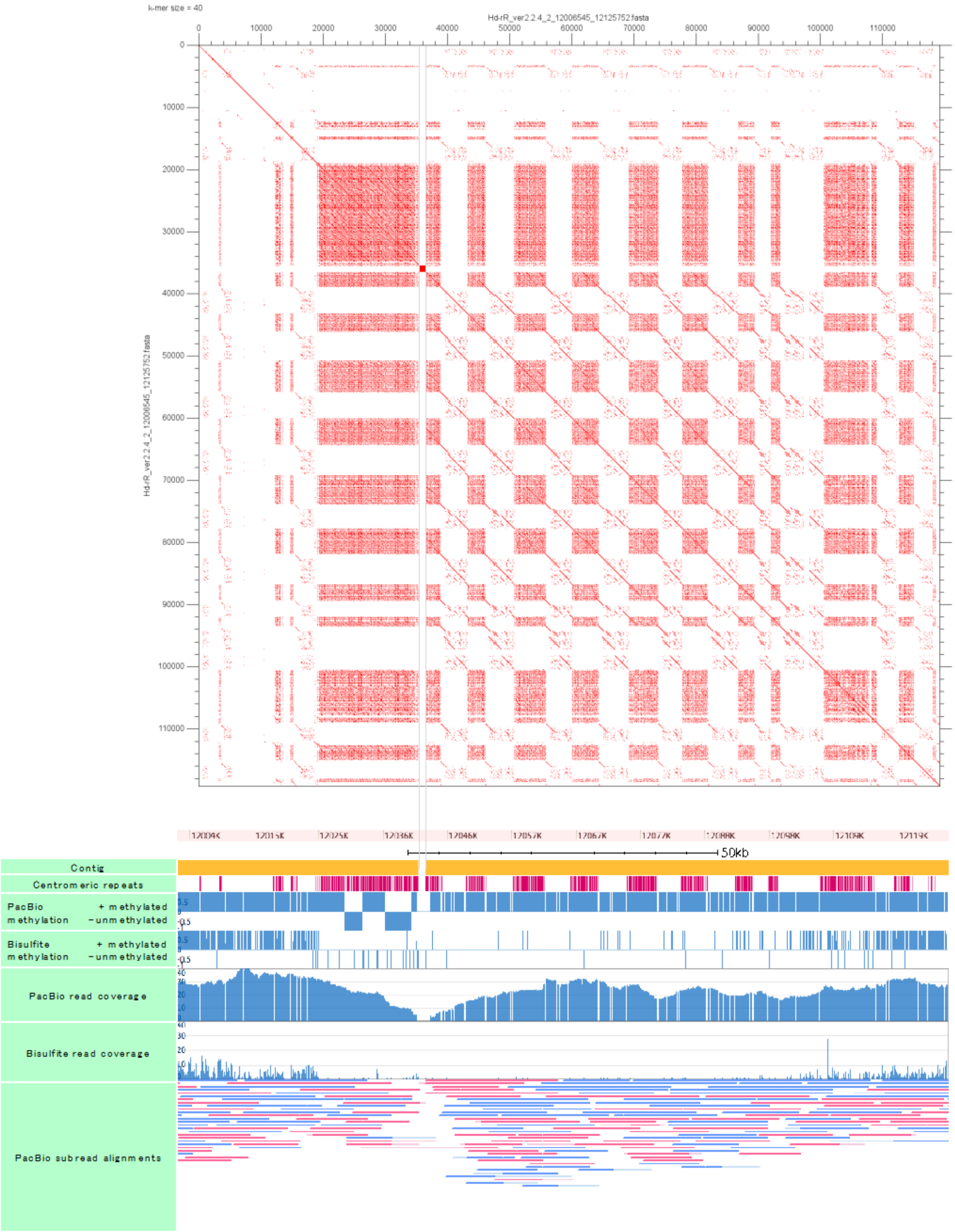

k-mer size = 40
Hd-rR_ver2.2.4_2_12006545_12125752.fasta
Hd-rR_ver2.2.4_2_12006545_12125752.fasta
50kb
Contig
Centromeric repeats
PacBio methylation
+ methylated
- unmethylated
Bisulfite methylation
+ methylated
- unmethylated
PacBio read coverage
Bisulfite read coverage
PacBio subread alignments

Hd-rR chr.3

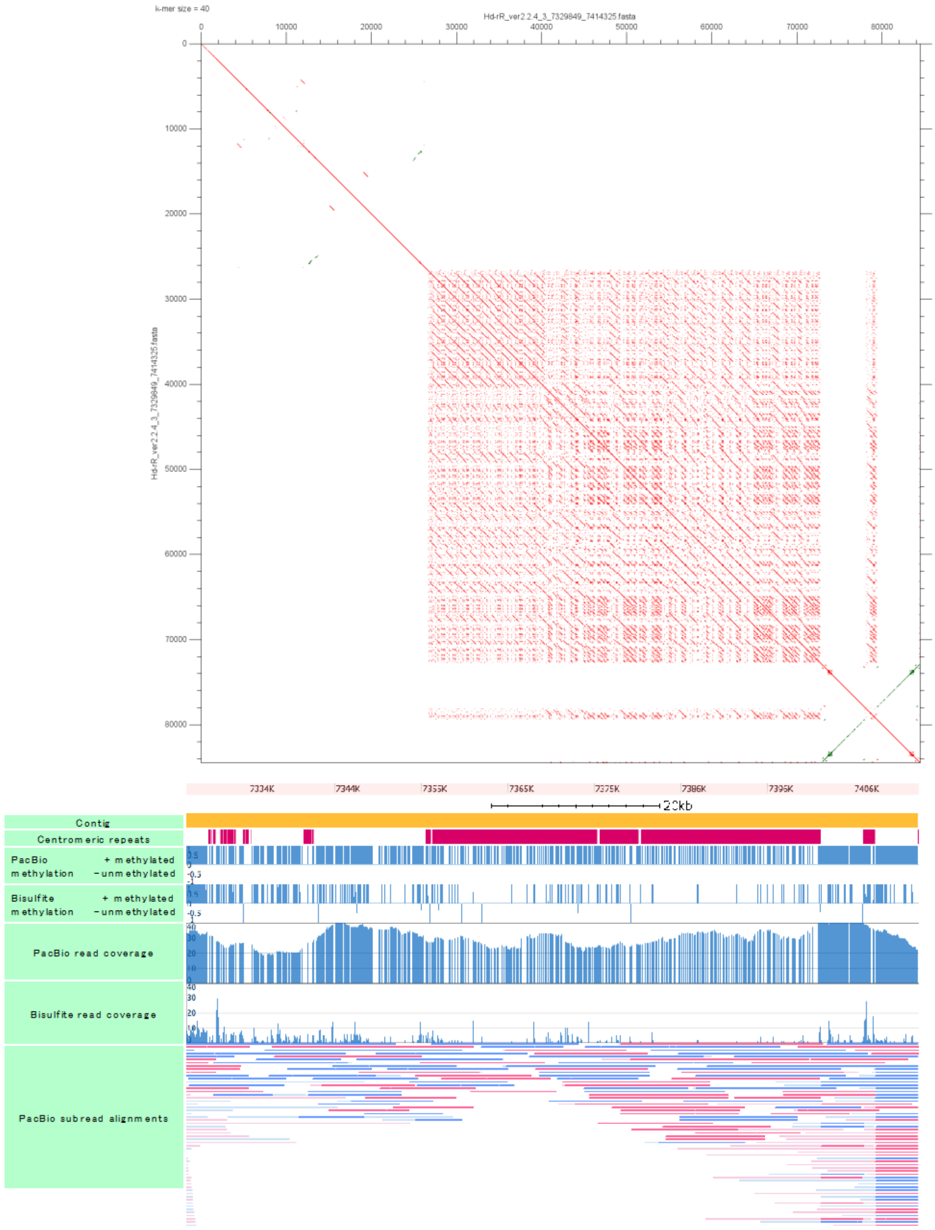
k-mer size = 40
Hd-rR_ver2.2.4_3_7329849_7414325.fasta
Contig
Centromeric repeats
PacBio methylation + methylated - unmethylated
Bisulfite methylation + methylated - unmethylated
PacBio read coverage
Bisulfite read coverage
PacBio subread alignments
20kb

Hd-rR chr.6

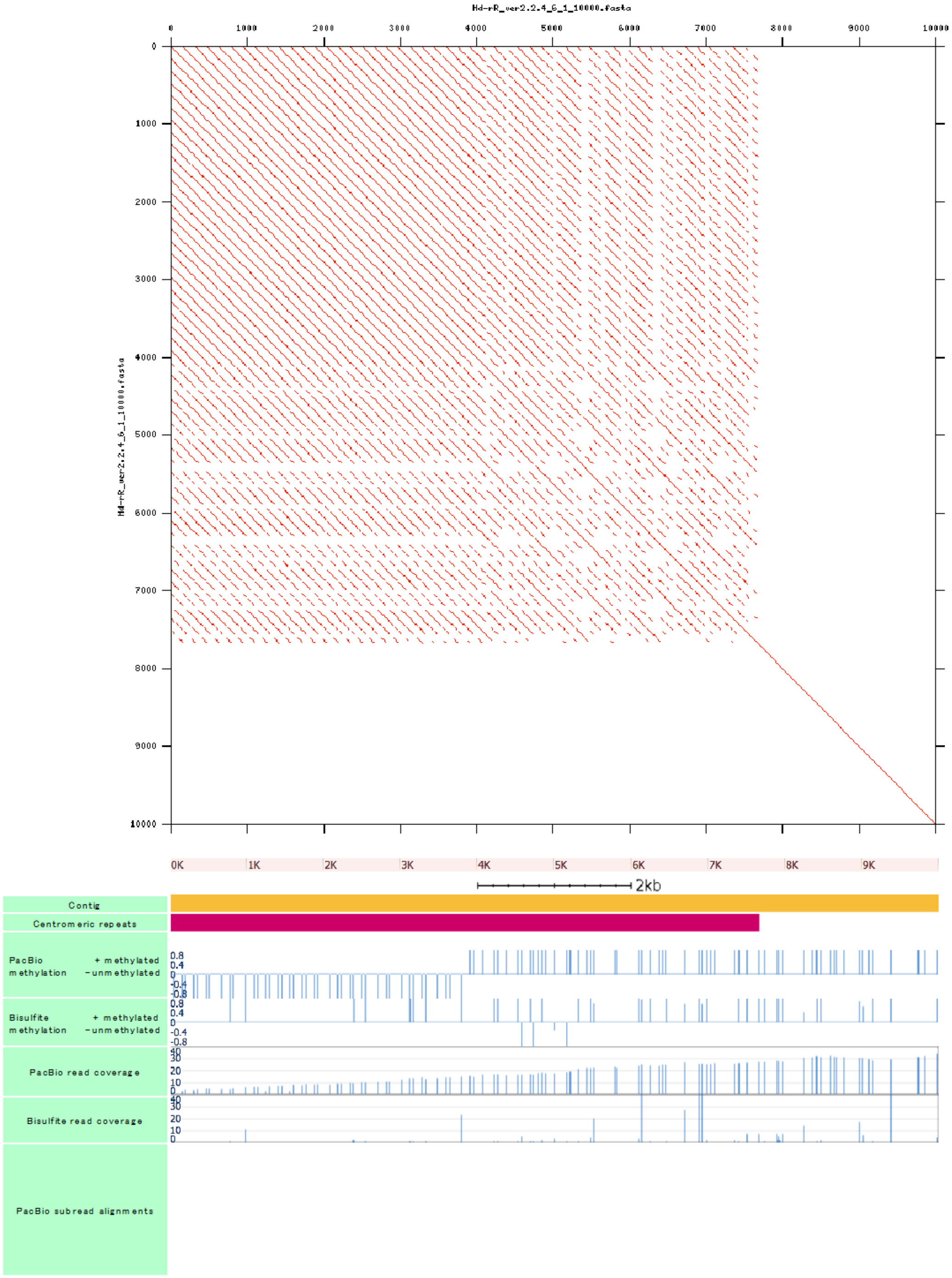

Hd-rR_ver2.2.4_6_1_10000.fasta
Contig
Centromeric repeats
PacBio methylation
+ methylated
- unmethylated
Bisulfite methylation
+ methylated
- unmethylated
PacBio read coverage
Bisulfite read coverage
PacBio subread alignments
2kb

Hd-rR chr.8

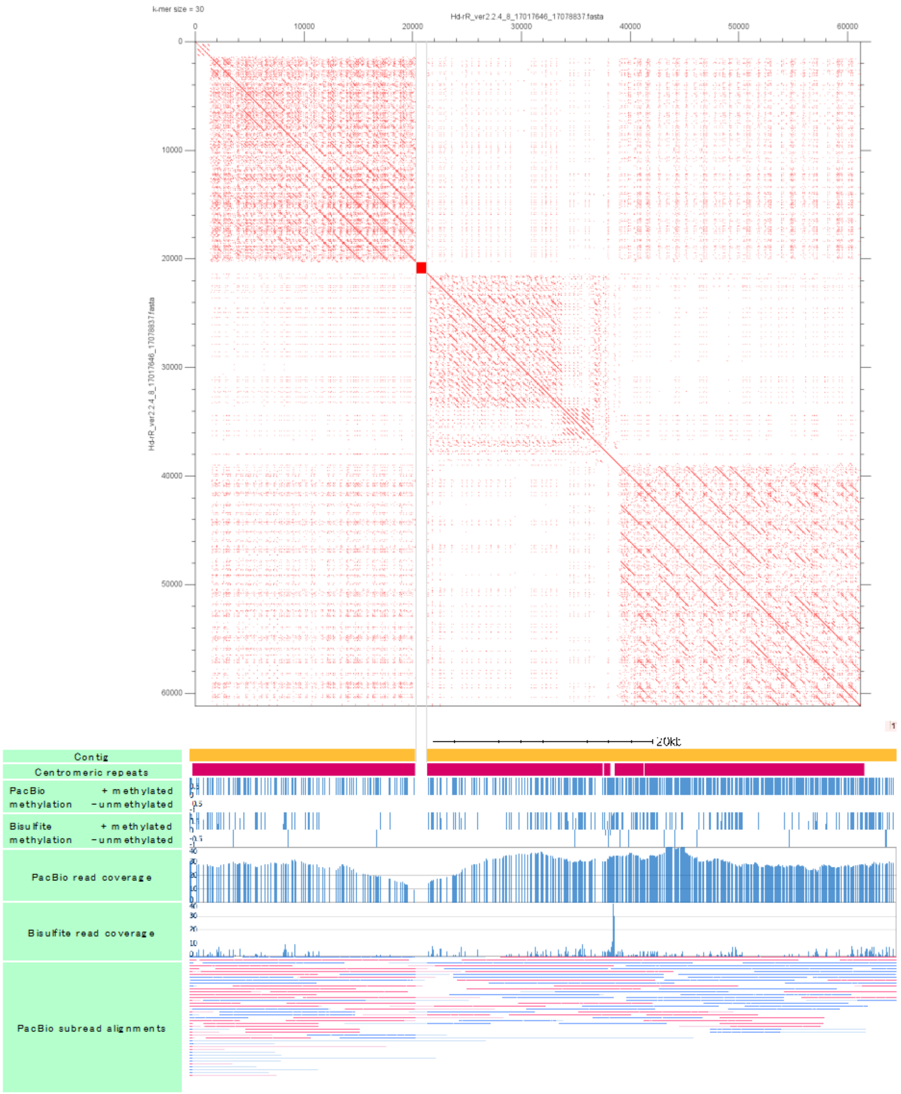

k-mer size = 30
Hd-rR_ver2.2.4_8_17017646_17078837.fasta
0
10000
20000
30000
40000
50000
60000
Hd-rR_ver2.2.4_8_17017646_17078837.fasta
20kb
Contig
Centromeric repeats
PacBio methylation
+ methylated
- unmethylated
Bisulfite methylation
+ methylated
- unmethylated
PacBio read coverage
Bisulfite read coverage
PacBio subread alignments

Hd-rR chr.9

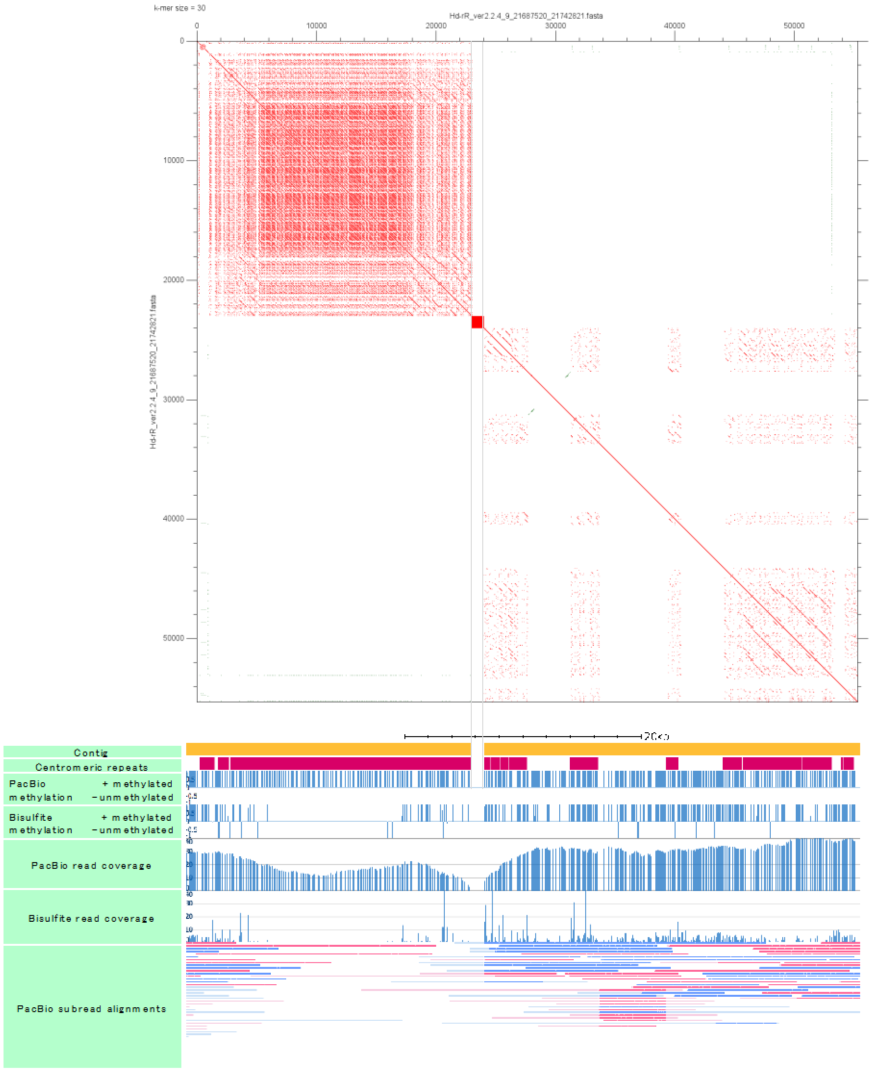
k-mer size = 30
Hd-rR_ver2.2.4_9_21687520_21742821.fasta
Contig
Centromeric repeats
PacBio methylation + methylated − unmethylated
Bisulfite methylation + methylated − unmethylated
PacBio read coverage
Bisulfite read coverage
PacBio subread alignments

Hd-rR chr.12

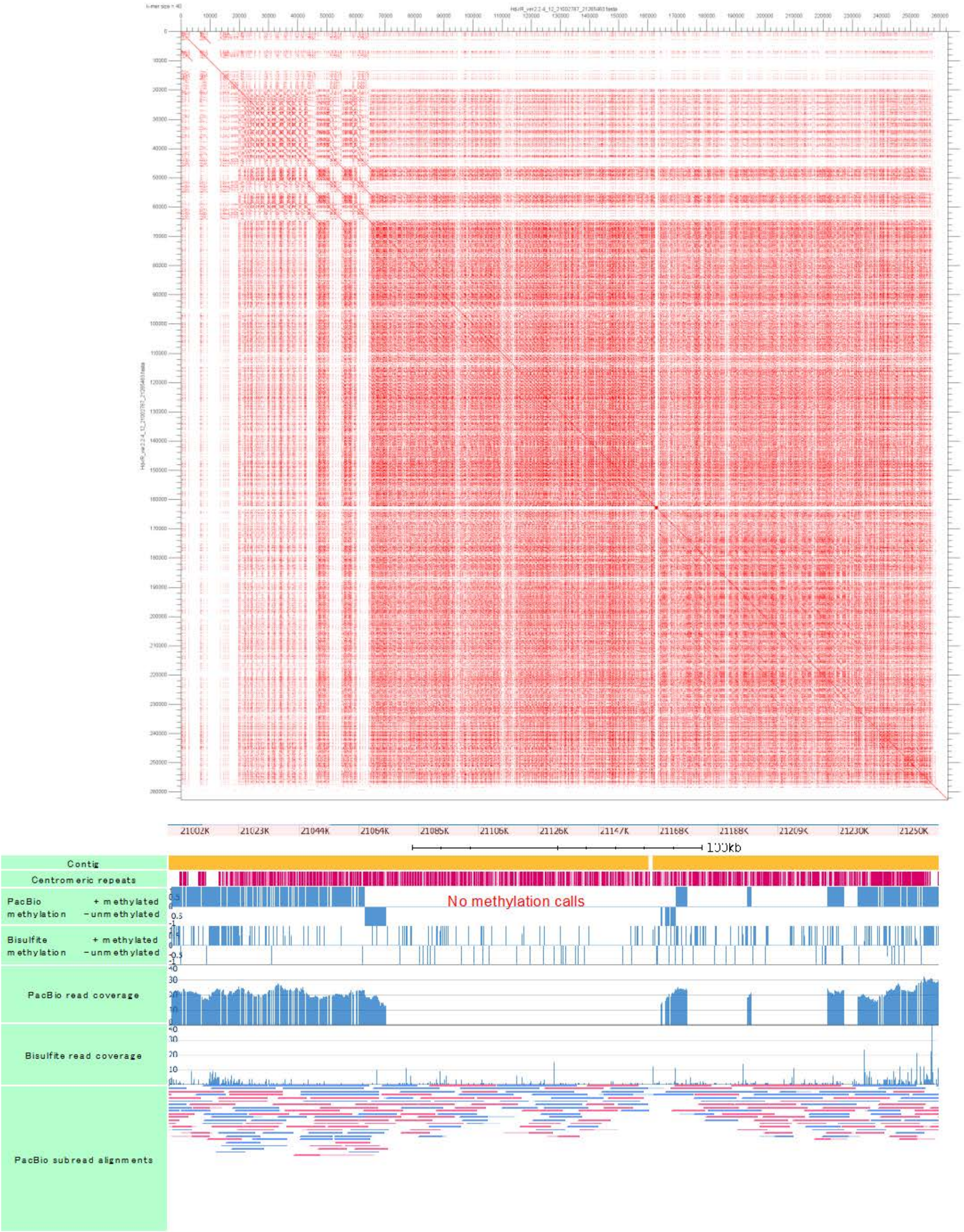
21002K
21023K
21044K
21064K
21085K
21106K
21126K
21147K
21168K
21188K
21209K
21230K
21250K
100kb
Contig
Centromeric repeats
PacBio methylation
+ methylated
− unmethylated
No methylation calls
Bisulfite methylation
+ methylated
− unmethylated
PacBio read coverage
Bisulfite read coverage
PacBio subread alignments

Hd-rR chr.13

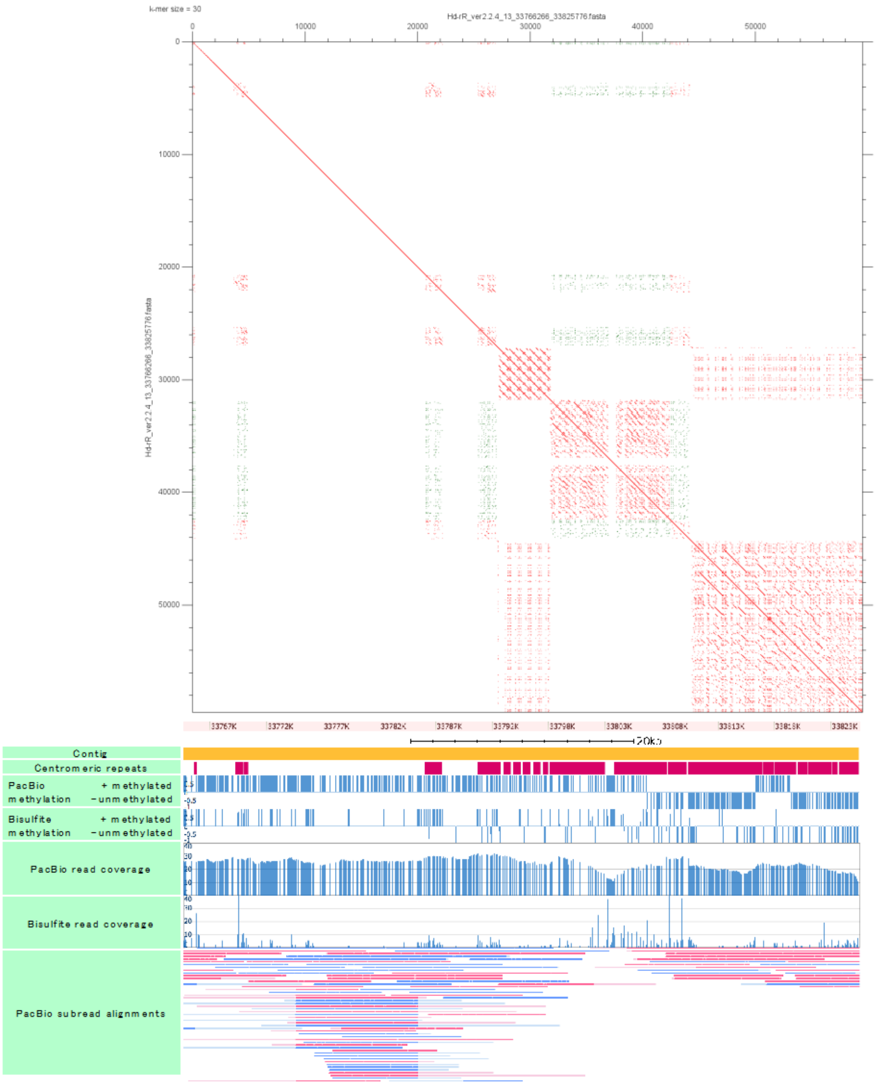
k-mer size = 30
Hd-rR_ver2.2.4_13_33766266_33825776.fasta
Contig
Centromeric repeats
PacBio methylation + methylated − unmethylated
Bisulfite methylation + methylated − unmethylated
PacBio read coverage
Bisulfite read coverage
PacBio subread alignments

Hd-rR chr.14

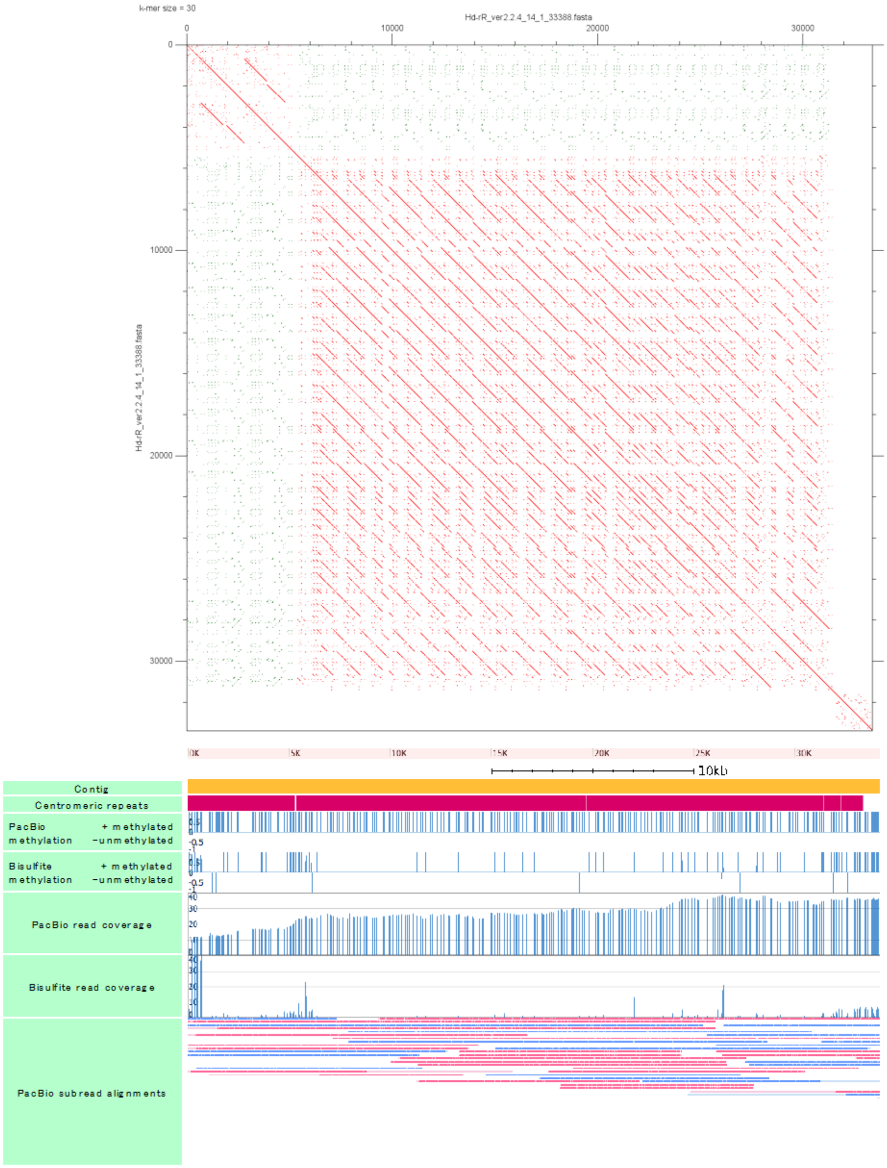
k-mer size = 30
Hd-rR_ver2.2.4_14_1_33388.fasta
0
10000
20000
30000
Hd-rR_ver2.2.4_14_1_33388.fasta
0K
5K
10K
15K
20K
25K
30K
10kb
Contig
Centromeric repeats
PacBio methylation
+ methylated
−unmethylated
Bisulfite methylation
+ methylated
−unmethylated
PacBio read coverage
Bisulfite read coverage
PacBio subread alignments

Hd-rR chr.18

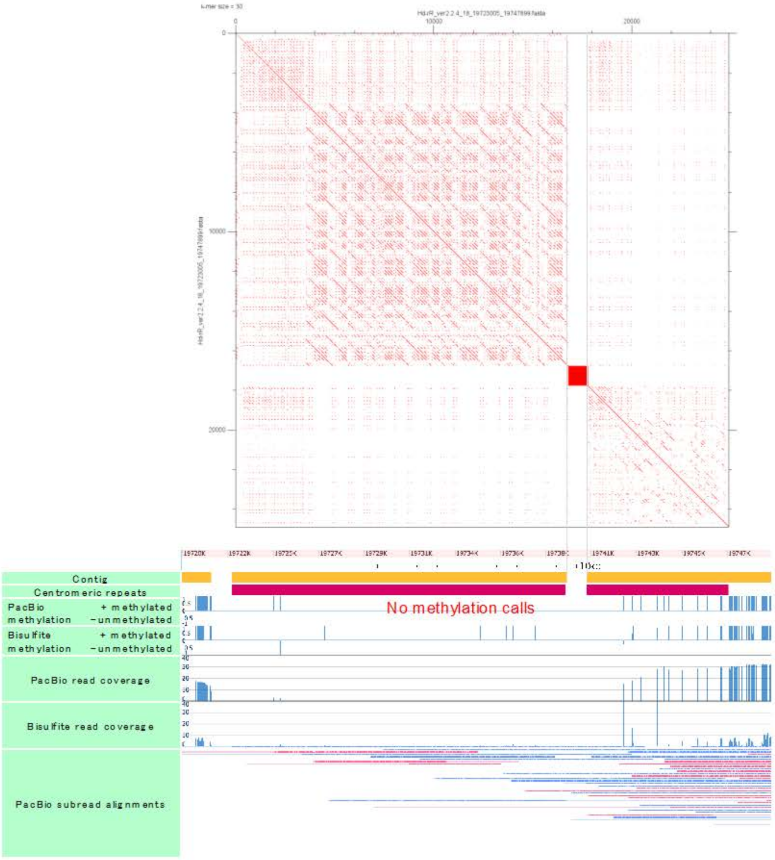

Contig
Centromeric repeats
PacBio methylation + methylated - unmethylated
Bisulfite methylation + methylated - unmethylated
No methylation calls
PacBio read coverage
Bisulfite read coverage
PacBio subread alignments

Hd-rR chr.19

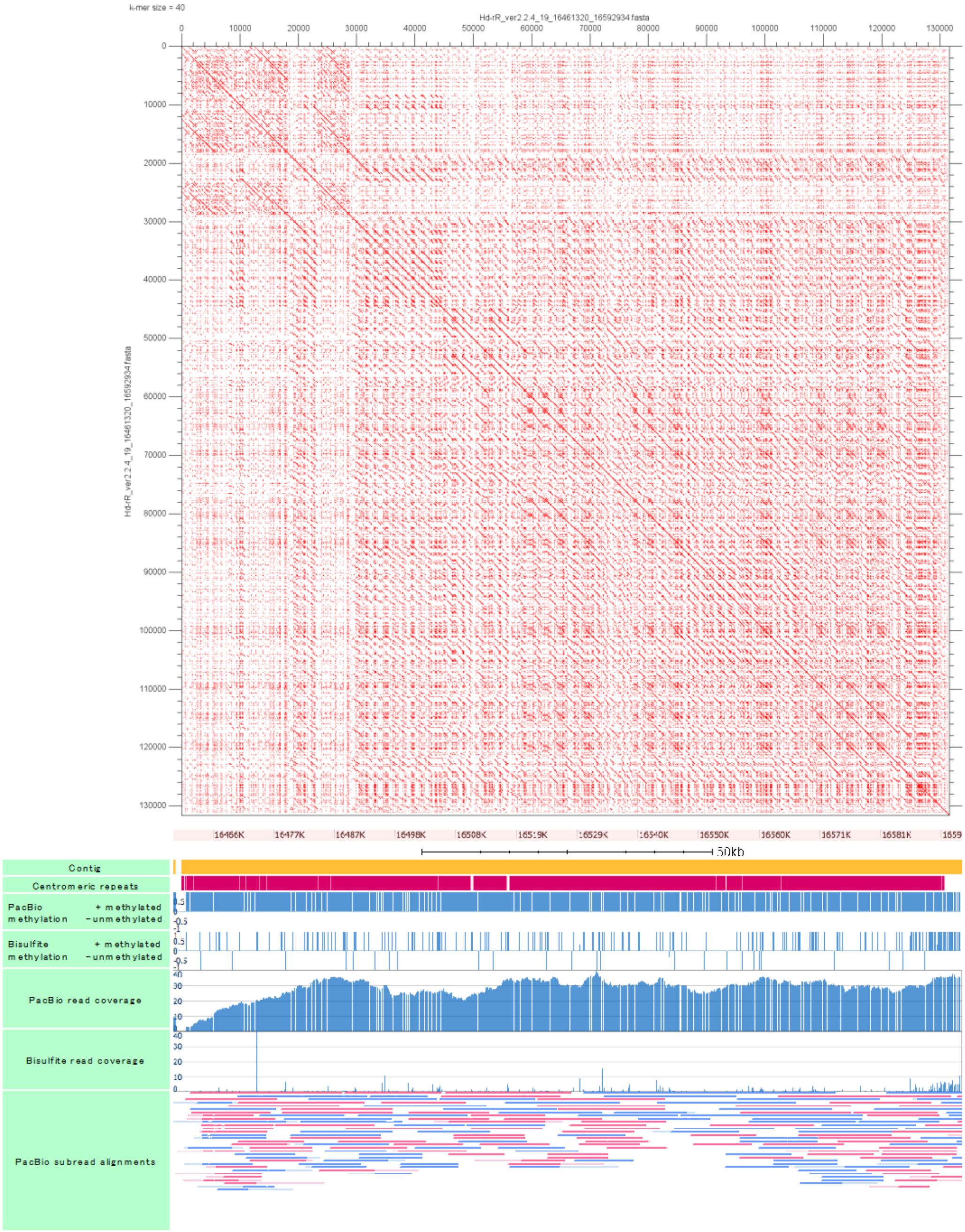
k-mer size = 40
Hd-rR_ver2.2.4_19_16461320_16592934.fasta
Contig
Centromeric repeats
PacBio methylation + methylated - unmethylated
Bisulfite methylation + methylated - unmethylated
PacBio read coverage
Bisulfite read coverage
PacBio subread alignments
50kb

Hd-rR chr.20

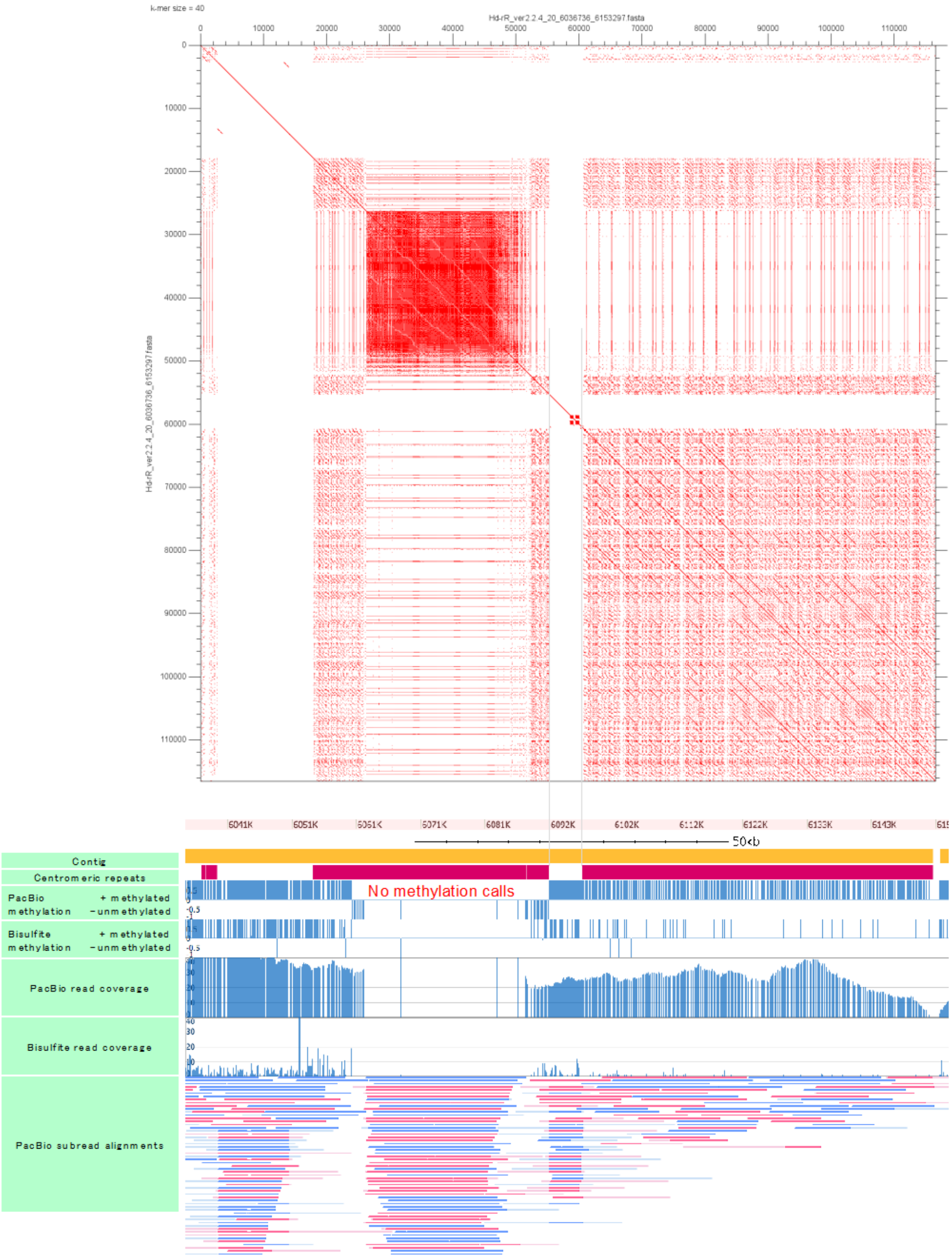

k-mer size = 40
Hd-rR_ver2.2.4_20_6036736_6153297.fasta
No methylation calls
50kb
Contig
Centromeric repeats
PacBio methylation + methylated - unmethylated
Bisulfite methylation + methylated - unmethylated
PacBio read coverage
Bisulfite read coverage
PacBio subread alignments

Hd-rR chr.21

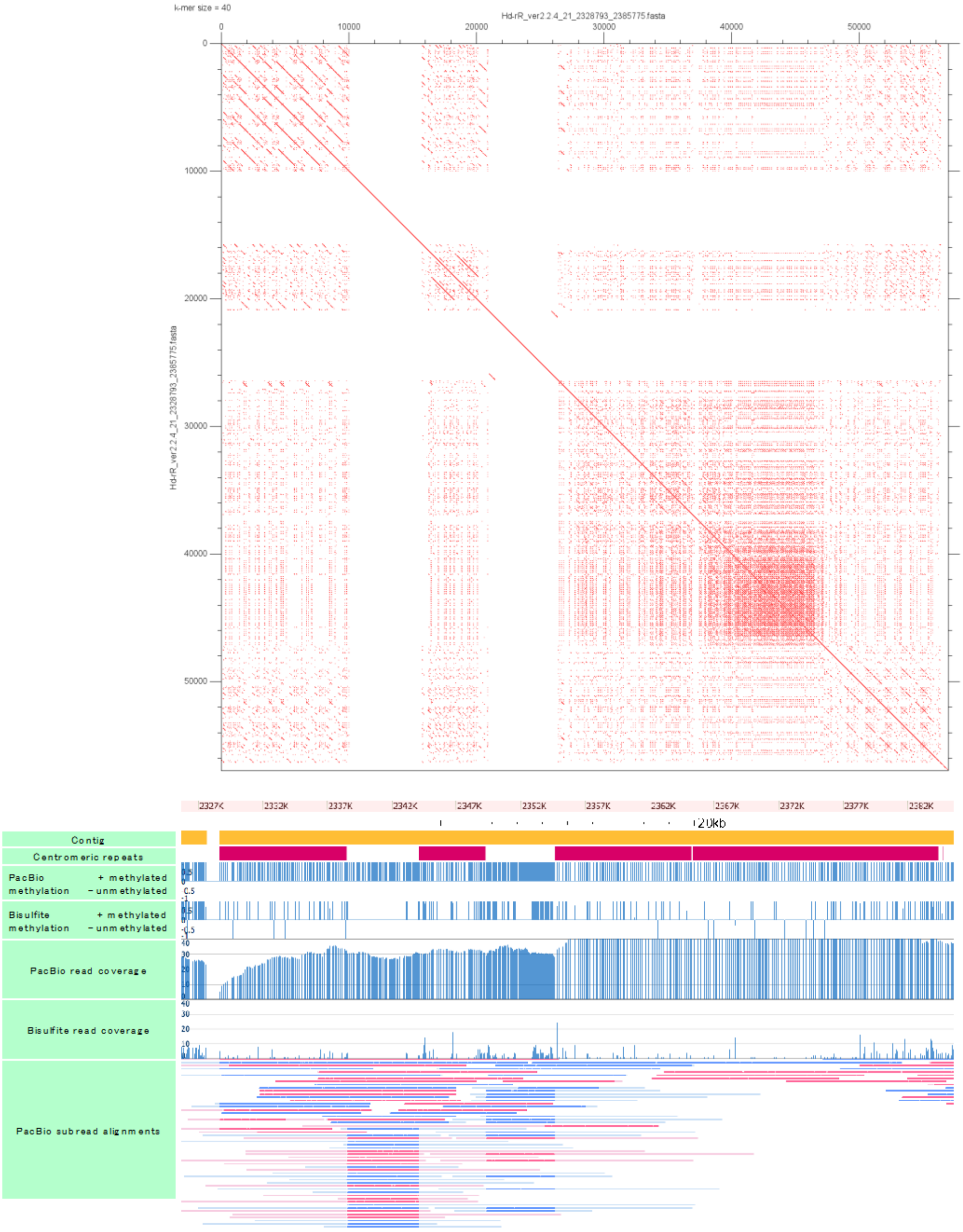

k-mer size = 40
Hd-rR_ver2.2.4_21_2328793_2385775.fasta
Contig
Centromeric repeats
PacBio methylation
+ methylated
− unmethylated
Bisulfite methylation
+ methylated
− unmethylated
PacBio read coverage
Bisulfite read coverage
PacBio subread alignments
20kb

Hd-rR chr.22

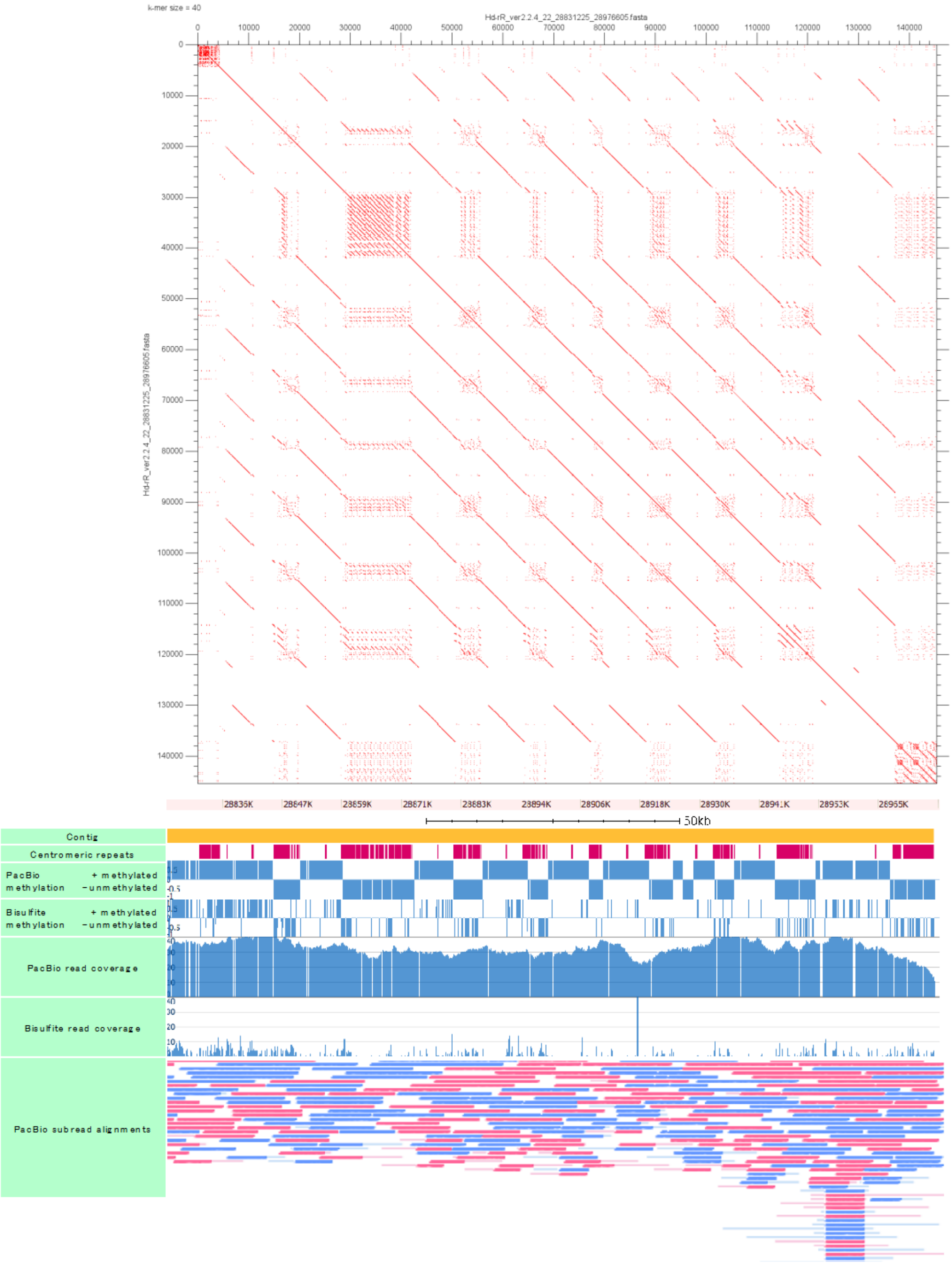

k-mer size = 40
Hd-rR_ver2.2.4_22_28831225_28976605.fasta
Hd-rR_ver2.2.4_22_28831225_28976605.fasta
28835K
28847K
28859K
28871K
28883K
28894K
28906K
28918K
28930K
28941K
28953K
28965K
50kb
Contig
Centromeric repeats
PacBio methylation
+ methylated
− unmethylated
Bisulfite methylation
+ methylated
− unmethylated
PacBio read coverage
Bisulfite read coverage
PacBio subread alignments

HSOK chr.2

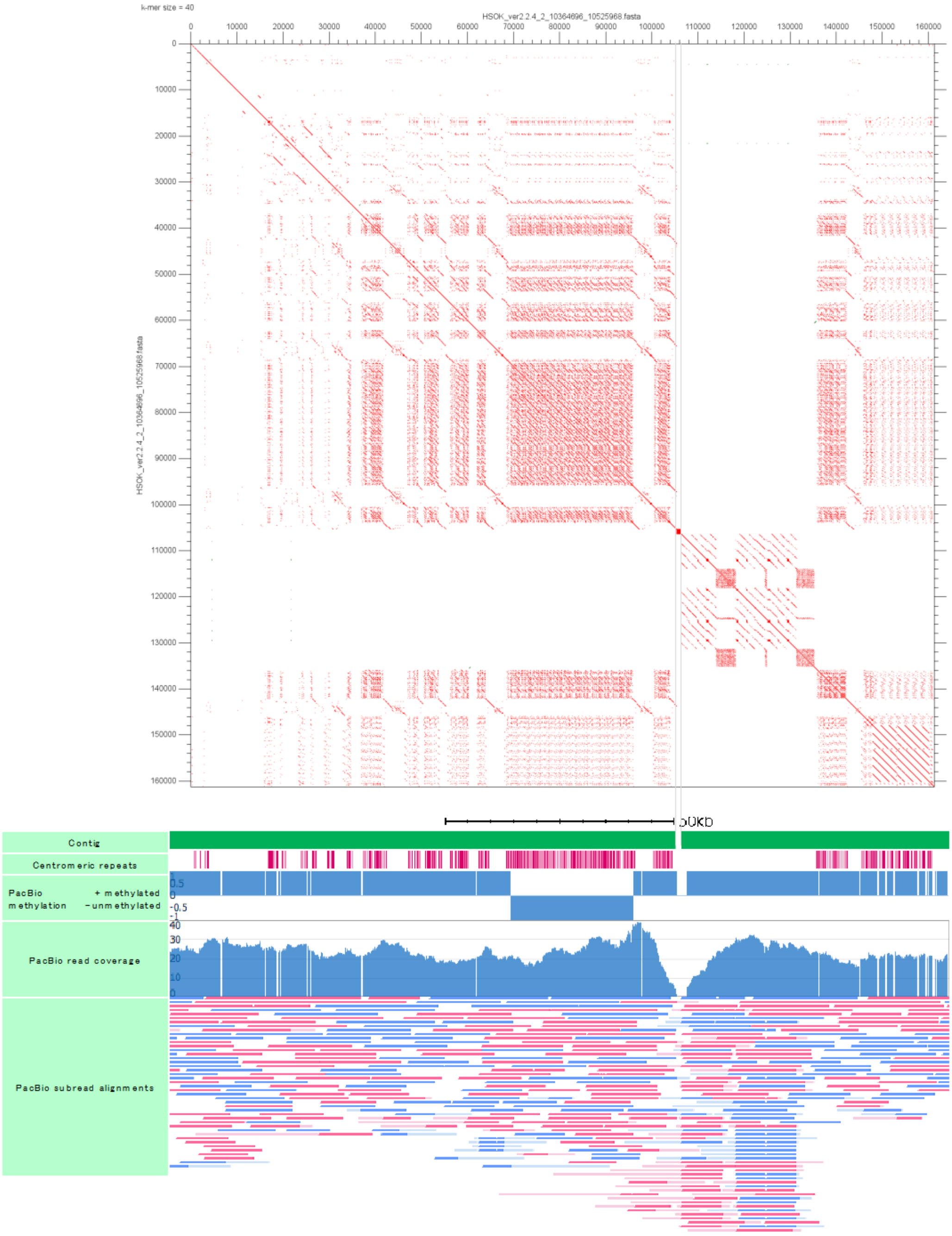

k-mer size = 40
HSOK_ver2.2.4_2_10364696_10525968.fasta
0
10000
20000
30000
40000
50000
60000
70000
80000
90000
100000
110000
120000
130000
140000
150000
160000
HSOK_ver2.2.4_2_10364696_10525968.fasta
50kb
Contig
Centromeric repeats
PacBio methylation
+ methylated
− unmethylated
0.5
0
-0.5
-1
PacBio read coverage
40
30
20
10
0
PacBio subread alignments

HSOK chr.4

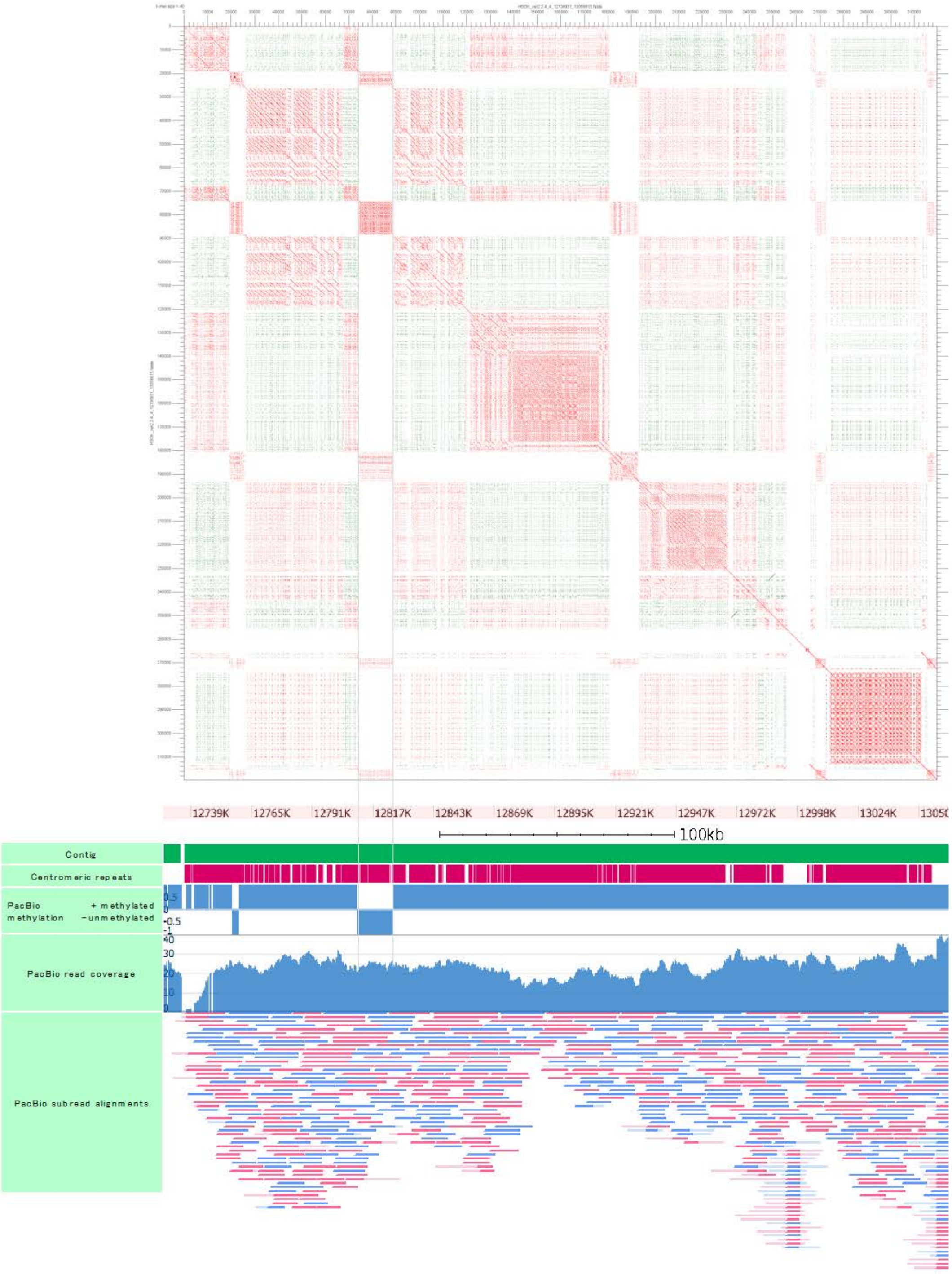

12739K
12765K
12791K
12817K
12843K
12869K
12895K
12921K
12947K
12972K
12998K
13024K
13050
100kb
Contig
Centromeric repeats
PacBio methylation
+ methylated
− unmethylated
0.5
0
-0.5
-1
40
30
20
10
0
PacBio read coverage
PacBio subread alignments

HSOK chr.7

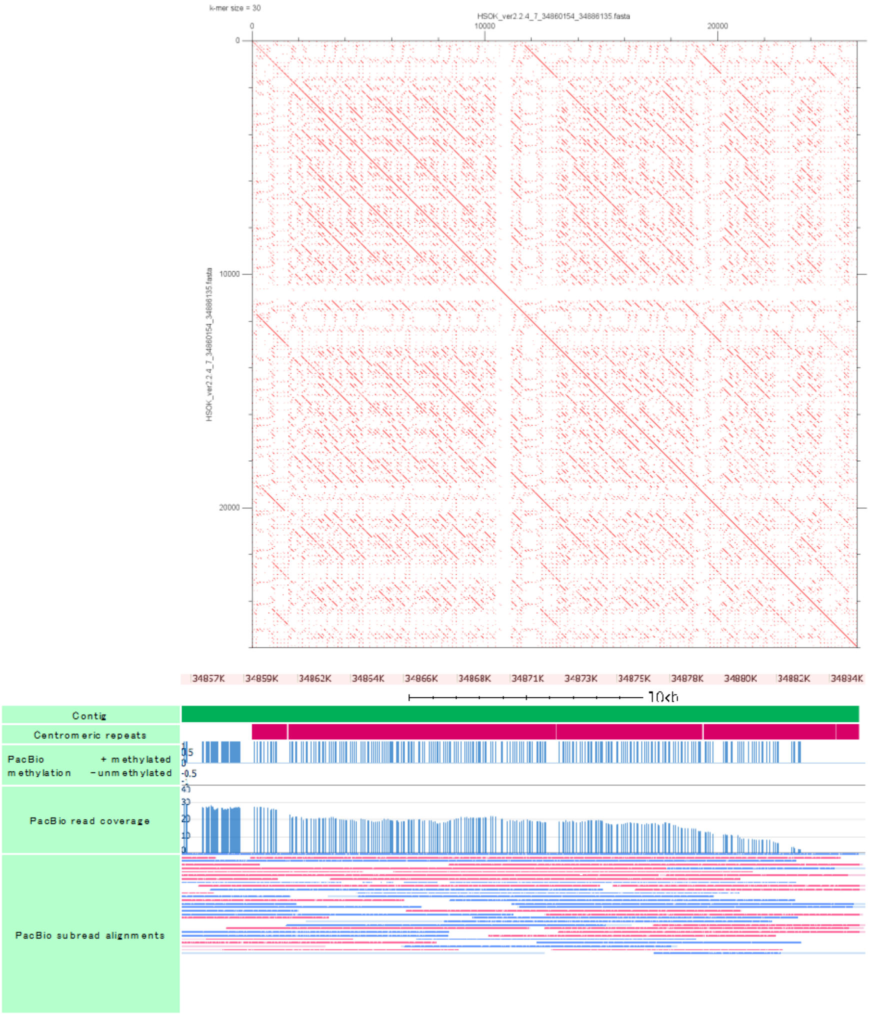

k-mer size = 30
HSOK_ver2.2.4_7_34860154_34886135.fasta
0
10000
20000
34857K
34859K
34862K
34864K
34866K
34868K
34871K
34873K
34875K
34878K
34880K
34882K
34884K
10kb
Contig
Centromeric repeats
PacBio methylation
+ methylated
− unmethylated
0.5
0
-0.5
30
20
10
0
PacBio read coverage
PacBio subread alignments

HSOK chr.8

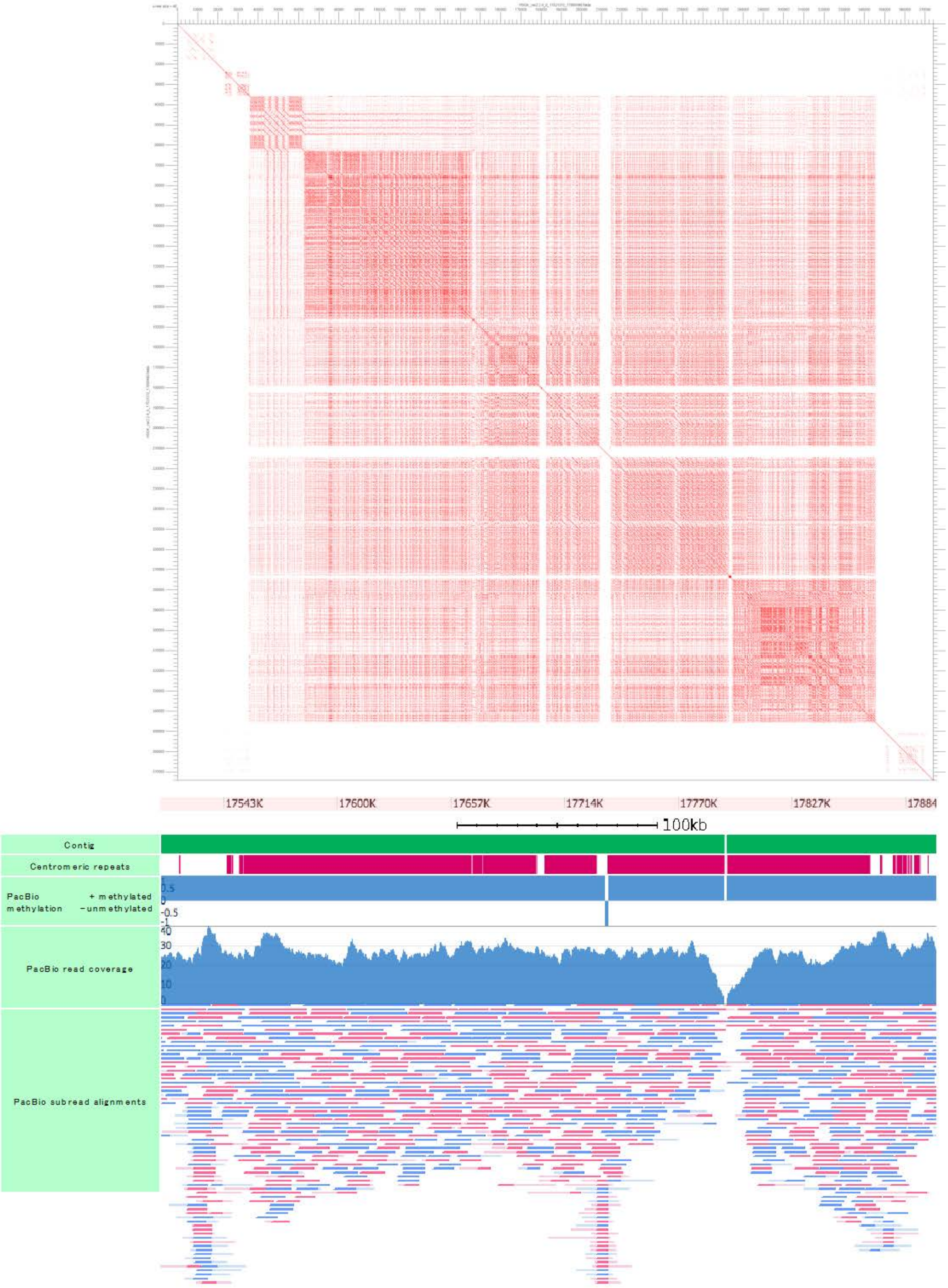

17543K
17600K
17657K
17714K
17770K
17827K
17884
100kb
Contig
Centromeric repeats
PacBio methylation
+ methylated
- unmethylated
PacBio read coverage
PacBio subread alignments

HSOK chr.11

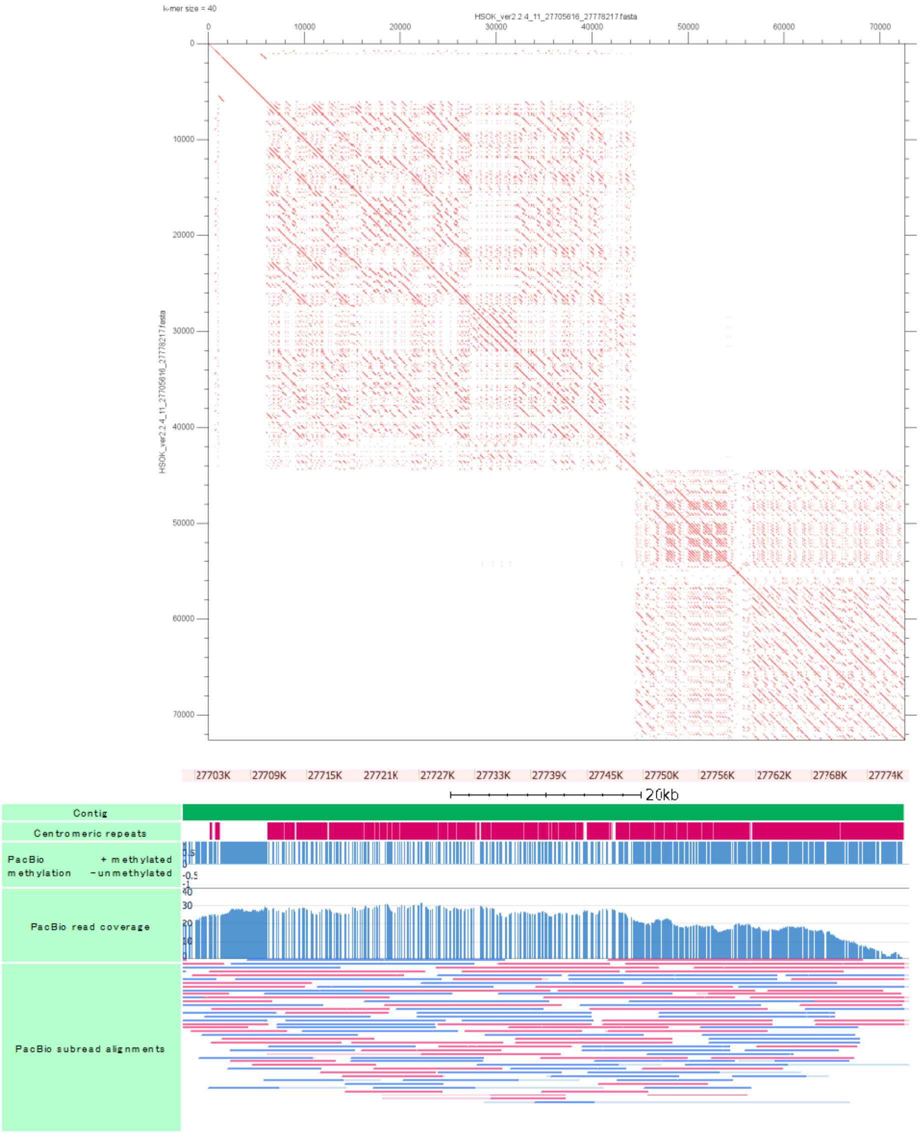
k-mer size = 40
HSOK_ver2.2.4_11_27705616_27778217.fasta
0
10000
20000
30000
40000
50000
60000
70000
27703K
27709K
27715K
27721K
27727K
27733K
27739K
27745K
27750K
27756K
27762K
27768K
27774K
20kb
Contig
Centromeric repeats
PacBio methylation
+ methylated
- unmethylated
PacBio read coverage
PacBio subread alignments

HSOK chr.12

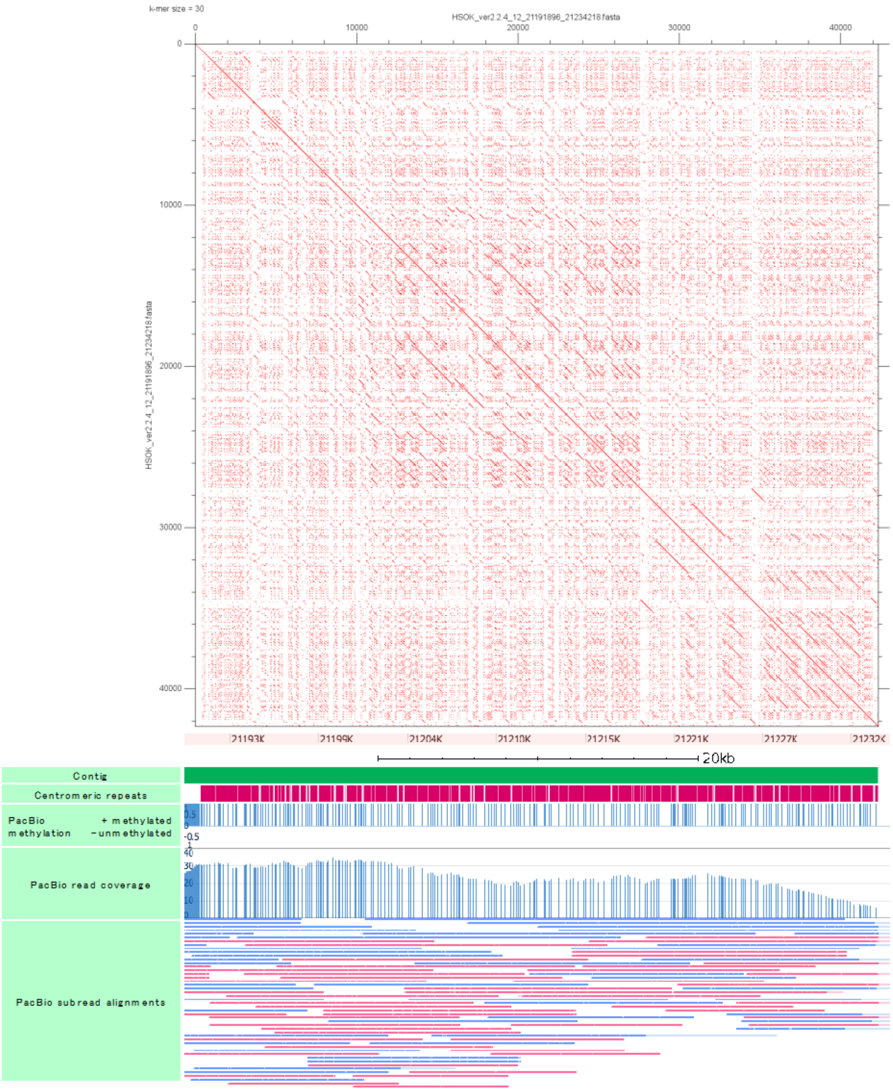
k-mer size = 30
HSOK_ver2.2.4_12_21191896_21234218.fasta
0
10000
20000
30000
40000
HSOK_ver2.2.4_12_21191896_21234218.fasta
21193K
21199K
21204K
21210K
21215K
21221K
21227K
21232K
20kb
Contig
Centromeric repeats
PacBio methylation
+ methylated
- unmethylated
PacBio read coverage
PacBio subread alignments

HSOK chr.15

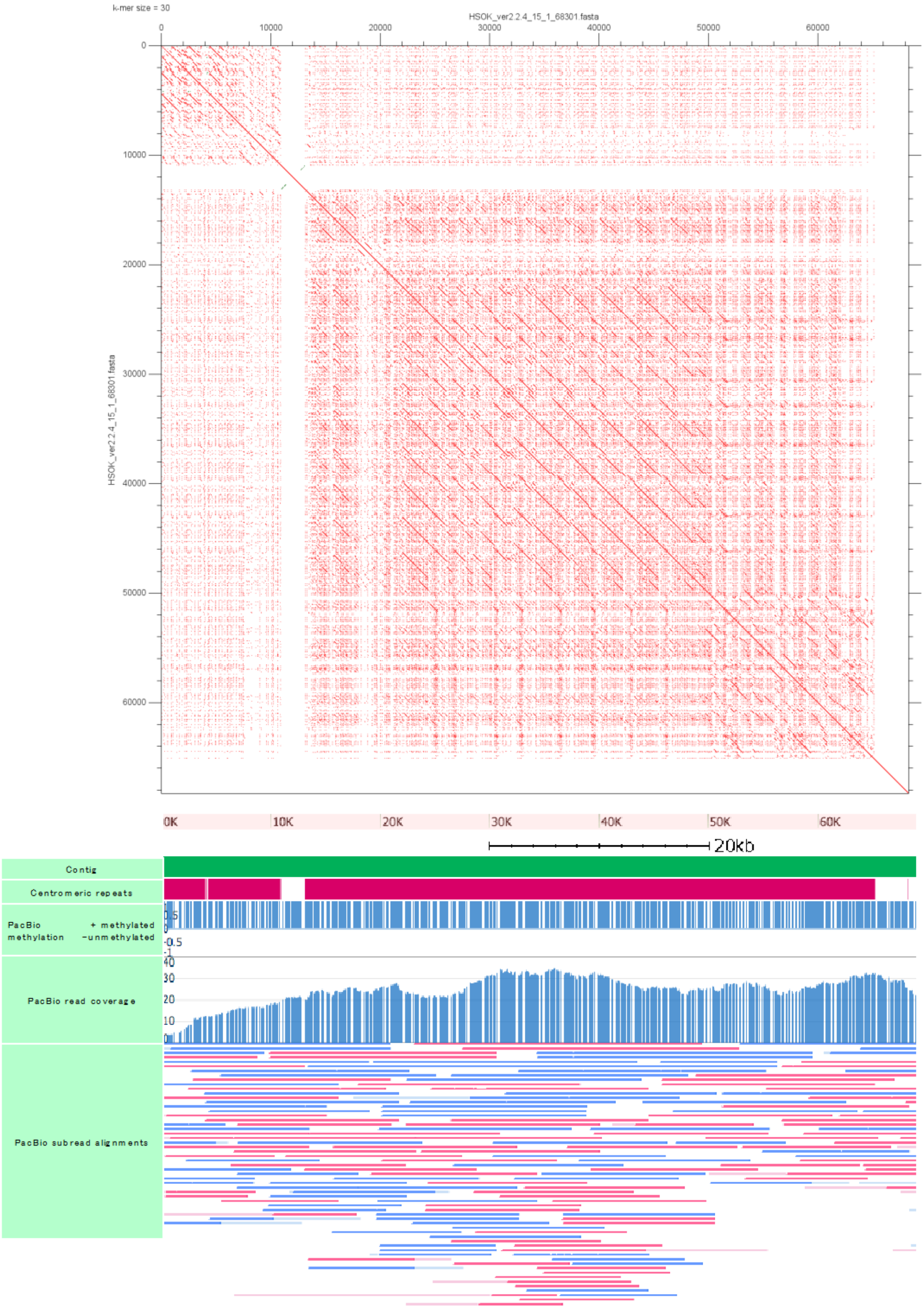
k-mer size = 30
HSOK_ver2.2.4_15_1_68301.fasta
0
10000
20000
30000
40000
50000
60000
HSOK_ver2.2.4_15_1_68301.fasta
0K
10K
20K
30K
40K
50K
60K
20kb
Contig
Centromeric repeats
PacBio methylation
+ methylated
−unmethylated
0.5
0
-0.5
-1
PacBio read coverage
40
30
20
10
0
PacBio subread alignments

HSOK chr.20

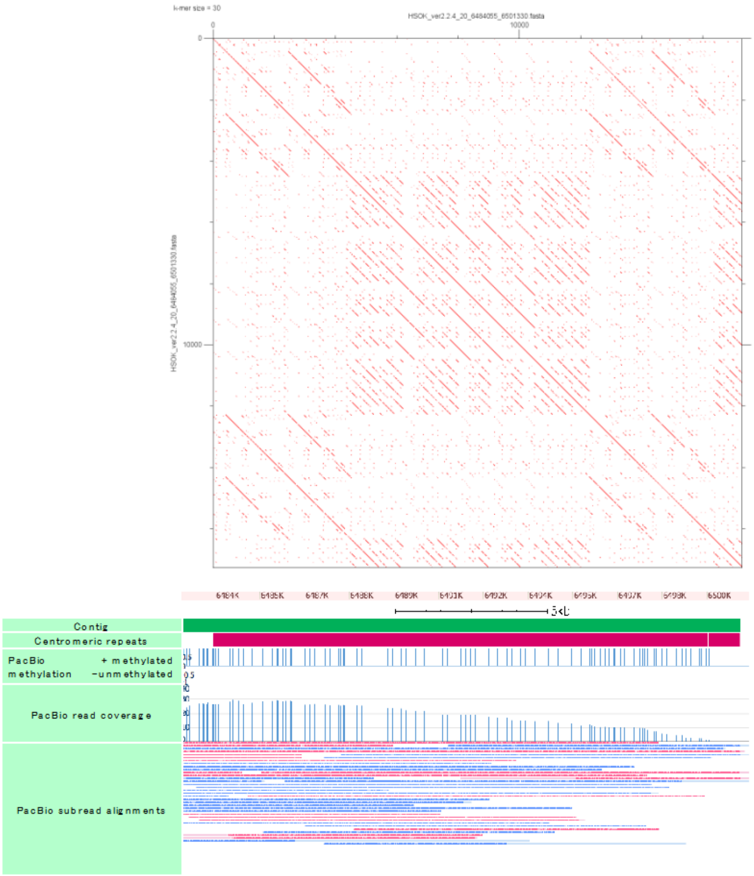
k-mer size = 30
HSOK_ver2.2.4_20_6484055_6501330.fasta
0
10000
HSOK_ver2.2.4_20_6484055_6501330.fasta
0
10000
Contig
Centromeric repeats
PacBio methylation
+ methylated
- unmethylated
PacBio read coverage
PacBio subread alignments

HSOK chr.23

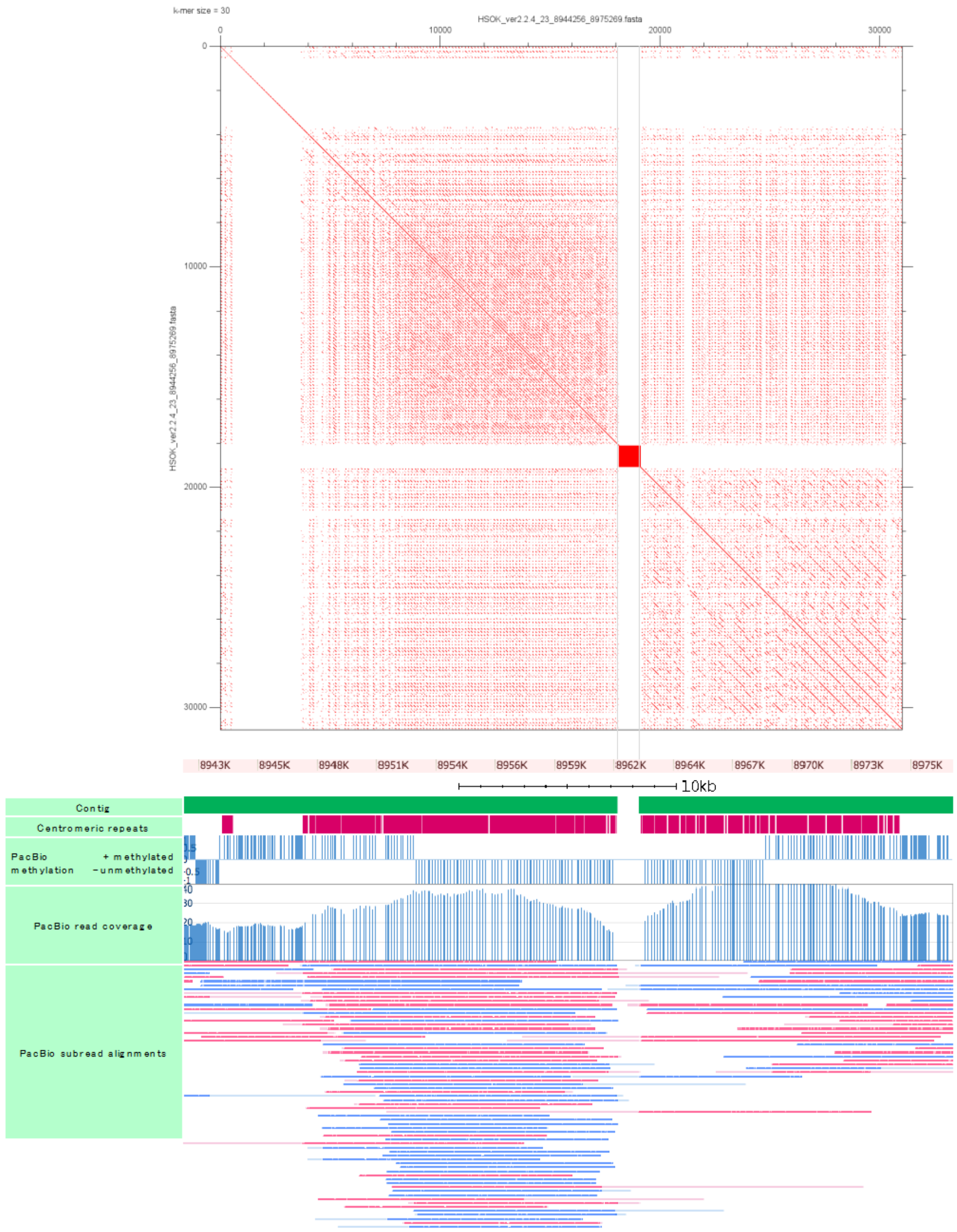

k-mer size = 30
HSOK_ver2.2.4_23_8944256_8975269.fasta
0
10000
20000
30000
HSOK_ver2.2.4_23_8944256_8975269.fasta
8943K
8945K
8948K
8951K
8954K
8956K
8959K
8962K
8964K
8967K
8970K
8973K
8975K
10kb
Contig
Centromeric repeats
PacBio methylation
+ methylated
− unmethylated
PacBio read coverage
PacBio subread alignments

Supplementary Figure 5. Validation of centromeric repeat regions and their CpG methylation states. We show regions with centromeric repeats on chromosomes of the Hd-rR and HSOK genomes. (Top) Dot plot of centromeric regions, where each dot represents 30- or 40-mer sequence match (indicated at the top left in each figure). Red and green dots indicate forward and reverse matches, respectively. Red blocks indicate contigs gaps. We can observe multiple patterns of higher order repeats that are represented by lines parallel to the diagonal in most of chromosomes (Hd-rR chr. 1, 3, 6, 8, 9, 12, 13, 14, 18, 19, 20, 21, 22, HSOK chr.2, 4, 7, 8, 11, 12, 15, 20, 23), uncovering broad divergence in higher order repeats. (Bottom) Snapshots of genome browser in centromeric regions. The yellow bars represent Hd-rR contigs, green bars HSOK contigs, and red bars centromeric repeats. Below the track for centromeric repeats, we display tracks for regional methylation prediction from PacBio reads (+, methylated; -, unmethylated), CpG-wise methylation from bisulfite reads, coverage of PacBio reads, coverage of bisulfite reads, and PacBio subreads alignments (red, forward; blue, reverse) by using BLASR. As bisulfite data are unavailable for the HSOK genome, we generated two tracks for the methylation status calculated from PacBio subreads and for PacBio subread coverage at each CpG site. On chromosomes 12, 18, and 20 in the Hd-rR genome, we could not make methylation calls on centromeric repeats from PacBio subreads, and we labelled these regions with “No methylation calls” to distinguish them from unmethylated regions.

We observed hypomethylated centromeric repeats in six Hd-rR chromosomes (chr. 1, 2, 6, 12, 13, and 22) and three HSOK chromosomes (chr. 2, 4, and 23). The bottom track shows PacBio subreads mapped to the centromeric regions, where respective red and blue lines show alignments in the forward and backward strands, and light colored boxes are soft-clipped parts in reads that fail to be aligned to the genome. The coverage of PacBio subreads could be larger than the number of aligned subreads because we used a stringent condition on the alignment of PacBio subreads to the contigs (see Methods). The dot plots and genome browser snapshots are equally scaled. During the course of our analysis, we used two versions of genome assembly, version 2.2.3 and 2.2.4. Precisely, in version 2.2.4, we added 11 contigs to the Hd-rR genome, revised the orientations of 12, 13, and 2 contigs, and reordered 6, 14, and 2 contigs in the respective Hd-rR, HNI, and HSOK genomes. Finally we adjusted all analytical results to the genomic positions in version 2.2.4.

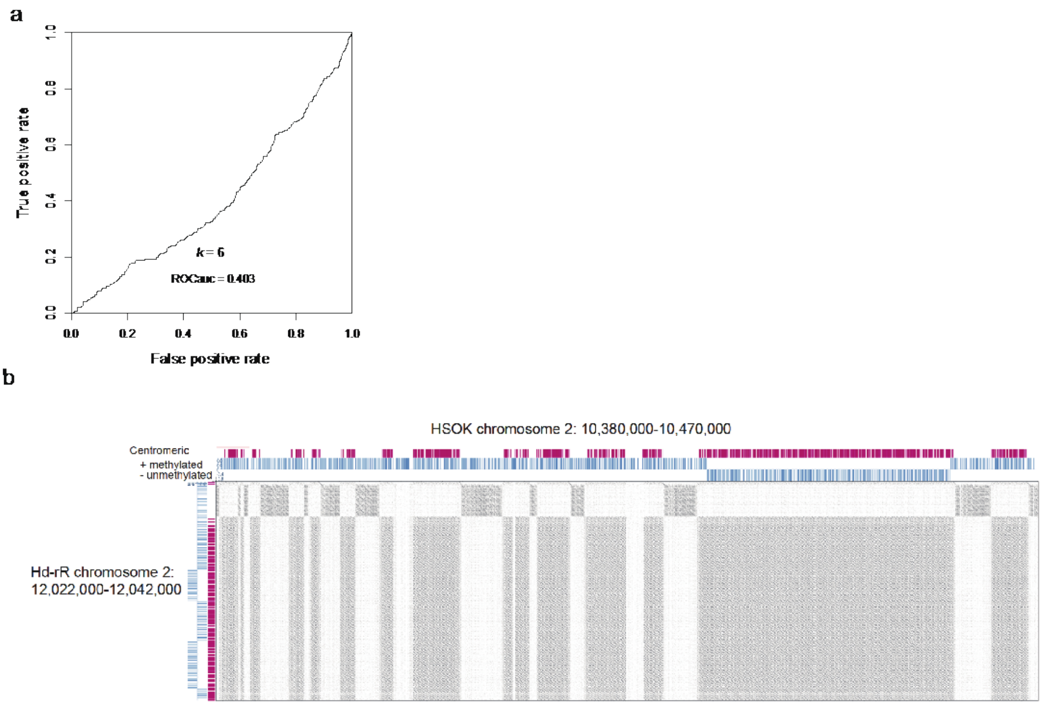

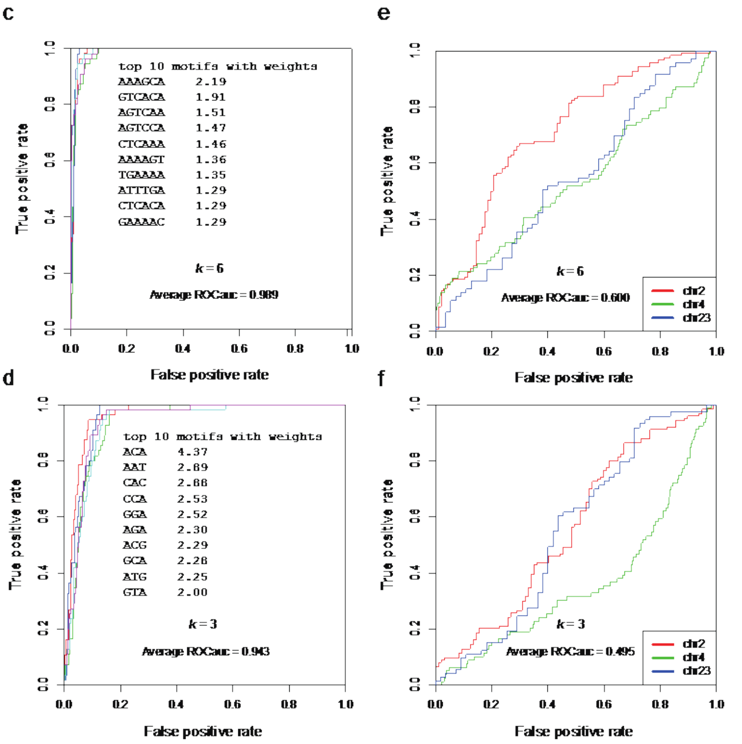

g

k-mer	Chr. 2	Chr. 4	Chr. 23
ACA	2.5	0.1	3.1
ATA	0.3	-0.2	2.3
CAC	1.0	0.9	2.3
ACG	0.9	2.3	1.8
CCA	1.8	0.3	1.7
GGA	1.2	1.2	1.6
GTA	1.5	0.5	1.6
AAA	0.6	1.3	1.5
ATG	1.0	-0.6	1.3
GAC	-0.4	2.2	1.1
AAC	1.5	0.8	0.9
ACT	0.3	0.9	0.5
AGC	1.7	-1.1	0.3
GCC	0.3	-0.8	0.1
AGC	-0.5	0.0	0.0
CTC	-1.0	-0.9	0.0
TAA	2.4	0.1	0.0
TCA	-0.7	-0.1	-0.3
CCC	0.4	0.5	-0.4
ATC	-1.0	-1.5	-0.4
AAC	0.2	0.8	0.6
CGC	-0.5	0.0	-0.7
CAA	0.5	-0.4	-0.7
CCA	-0.5	-0.4	-0.8
CAG	0.0	-0.7	-0.8
CTA	1.1	1.1	0.9
CCG	-0.8	-0.2	-1.1
ACC	-0.9	-0.3	-1.1
AGA	-1.4	-1.5	-1.2
GAA	1.1	0.7	1.4
GCA	-1.0	-1.2	-2.0
AAT	-1.8	-1.2	-2.2

Supplementary Figure 6: Performance of *k*-mer SVM to classify hypo-methylated domains (HMD) and methylated sequences on HSOK centromeric regions (chr2, 4, and 23).

a. An SVM with *k*-mers was trained using HMDs and methylated sequences in non-centromeric regions of Hd-rR, and HSOK centromeric sequences were used as the test data (Methods). The area under the ROC curve (ROCauc) shown in the graph is low, implying that relevant *k*-mers enriched in non-centromeres do not characterize HMDs in centromeric repeats.

b. To illustrate the property observed in the above figure (**a**), we show a dot plot between two syntenic centromeric repeat regions with unmethylated subregions in chromosome 2 of Hd-rR and HSOK (see the correspondence between genetic markers in Figure 1b). The identity ratio between the representative monomers of the centromeric repeats in the Hd-rR and HSOK chromosome 2 was 85.7% (Supplementary Table 21). The dot plot illustrates the difficulty in distinguishing the underlying sequence compositions of hypermethylated and hypomethylated centromeric regions.

c,d. We evaluated the classification by a five-fold cross validation. The data set was randomly partitioned into five subsets, and one subset was used as the test data and the rest were used as the training data. We set *k*=6 (**c**) and *k*=3 (**d**) for comparison because setting *k*=6 slightly overfitted the training data. Each curve colored differently represents one classification of five cross validation tests. An average ROCauc of five cross validation shown in the graph implies the presence of HMD-specific *k*-mers within centromeric repeats in the three HSOK chromosomes. The top 10 sequence motifs of length *k*=6 and *k*=3 are put into individual graphs (**c** and **d**).

e,f. The evaluation of the classification by using different chromosomes for the test and training data. Among chr 2, 4, and 23, two chromosomes were used as the training data and the remaining one was used as the test data. *k*=6 (b) and *k*=3 (c) were used for comparison. For each curve, the chromosome used as the test data is labelled in the graph legend. An average ROCauc of three classifications is indicated within the graph. Both of true positive and false positive rates are low, implying that the sequence compositions of HMDs differ in individual chromosomes.

g. The table shows all 3-mers with their weights that were used for the prediction in the graphs (**d** and **f**). To highlight the difference in the weights, high, medium, and low weights are colored red, white, and green, respectively.

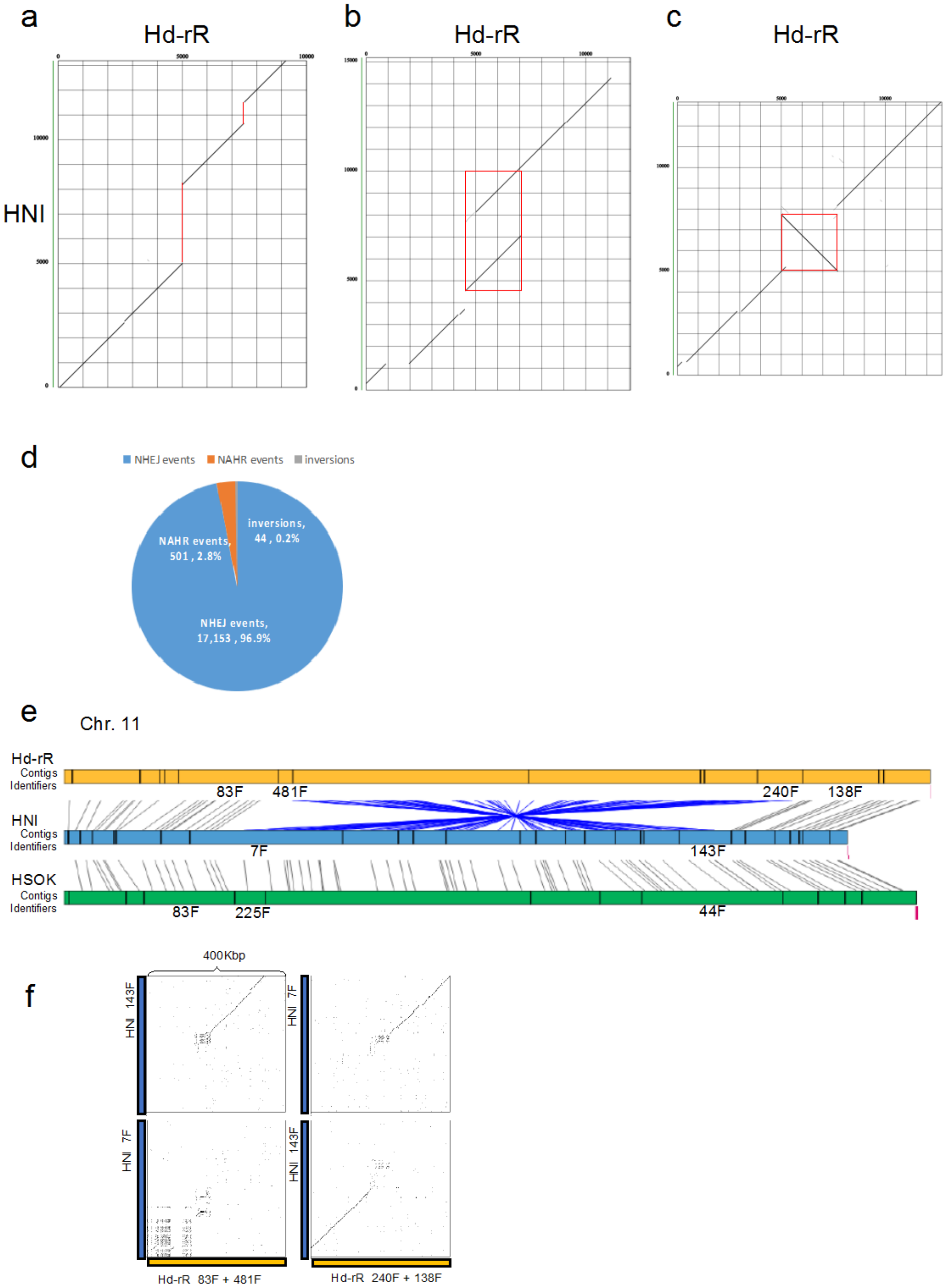
a
Hd-rR
HNI
b
Hd-rR
c
Hd-rR
d
NHEJ events
NAHR events
inversions
inversions, 44 , 0.2%
NAHR events, 501 , 2.8%
NHEJ events, 17,153 , 96.9%
e
Chr. 11
Hd-rR Contigs Identifiers
83F
481F
240F
138F
HNI Contigs Identifiers
7F
143F
HSOK Contigs Identifiers
83F
225F
44F
f
400Kbp
HNI 143F
HNI 7F
HNI 7F
HNI 143F
Hd-rR 83F + 481F
Hd-rR 240F + 138F

Supplementary Figure 7: Examples of large-scale structural variants.

We categorized mid-sized structural variants (SVs) into three classes:

a. Non-homologous end-joining (NHEJ) (including insertions and deletions);

b. Non-allelic homologous recombination (NAHR) such as duplications; and

c. Inversions.

d. Breakdown of mid-sized SVs that were identified between medaka Hd-rR and HNI strains. 96.9% if SVs were NHEJ events.

e. An extremely large inversion (>15 Mbp) in chromosome 11 was evident when Hd-rR and HNI were compared. The presence of the inversion was suggested by the Sanger-sequence genome assembly; however, the contigs assigned to chromosomes were not of sufficient length to reveal the boundaries of the inversion. In the present study, when we anchored contigs onto HNI and HSOK chromosome 11, we identified two pairs of contigs that had two sets of distal genetic markers that were separated by ~16Mb while we found no such pairs in Hd-rR, indicating that the inversion had occurred in the Hd-rR lineage. Contigs surrounding the breakpoints of the inversion are associated with their contig identifiers (*e.g.*, 83F and 481F). In the HNI genome, the two breakpoints are located at 7F and 143F, whereas in the Hd-rR genome, one breakpoint lies between 83F and 481F, and the other is between 240F and 138F. This is partly because the breakpoints lie in the long repetitive regions shown in Figure f.

f. We determined the inversion breakpoints in focal HNI and HSOK contigs by aligning these contigs with the corresponding region of Hd-rR. Dot plots comparing the four pairs of Hd-rR and HNI regions that contain the two breakpoints of the inversion. The inversion was surrounded by highly repetitive regions of ~200 kb and ~10 kb in size, which were difficult to detect using short read sequencing technology.

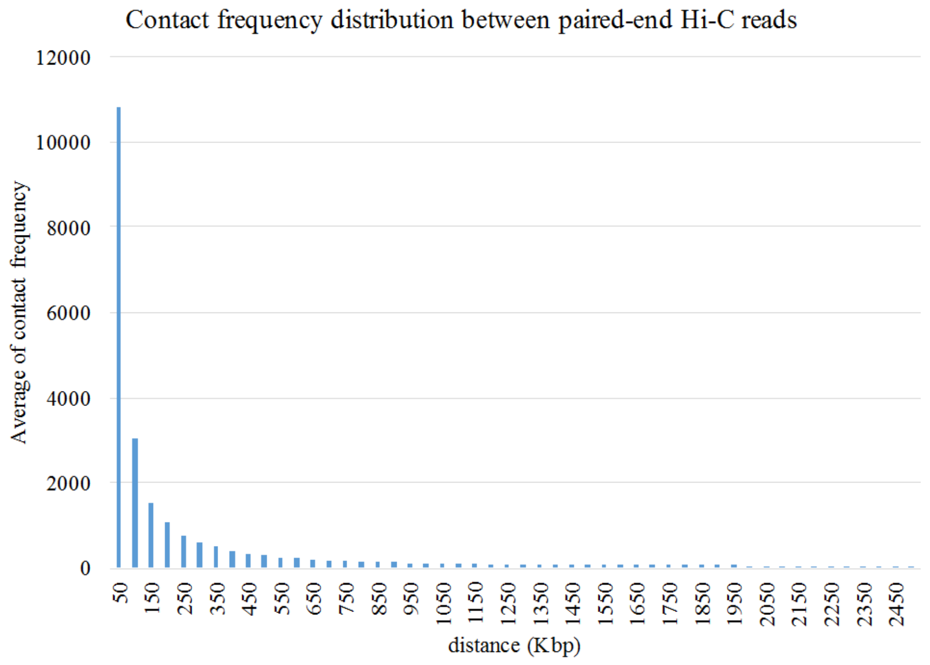

Supplementary Figure 8: Contact frequency distribution between paired-end Hi-C reads.

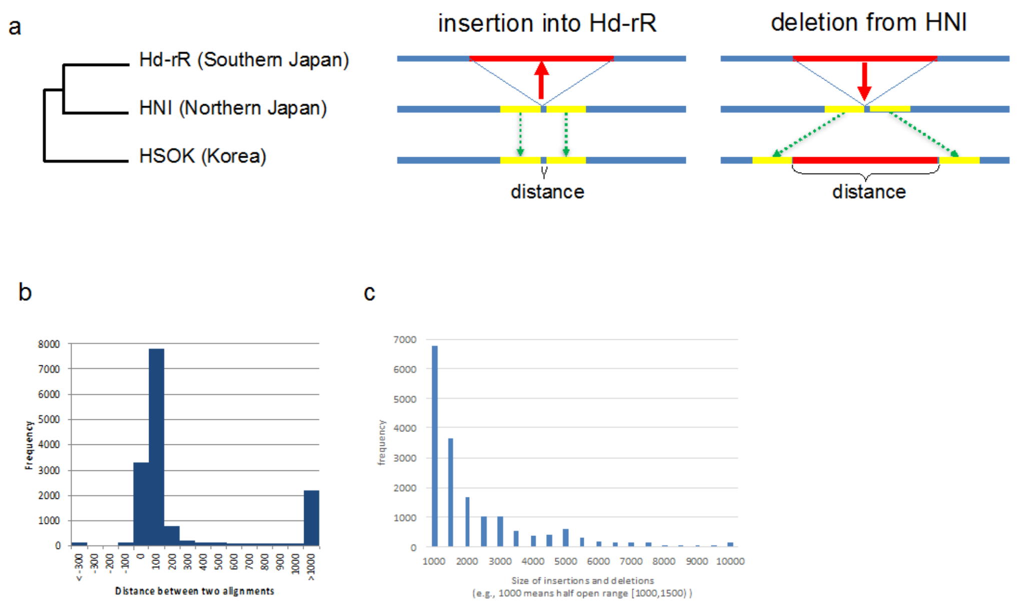

Supplementary Figure 9: Checking whether an insertion or a deletion occurred.

a. We used HSOK as an outgroup of Hd-rR and HNI determine whether a given event was an insertion into, or a deletion from, the focal genome. We mapped (to HSOK) the two 2,500-bp regions upstream and downstream from the positions in HNI (or Hd-rR, respectively) where the insertion/deletion events had occurred in Hd-rR (HNI). We measured the distance between the alignments of the two 2,500-bp regions in the HSOK genome.

b. The histogram shows the frequency distribution of these distances, and exhibits two peaks around 0 and >1000. As the two peaks were thus clearly separated, we classified events using the heuristic whereby events in the peak around 0 were insertions, and those around the other peak were deletions. The peak around 0 is broad because the three strains collected mutations during evolution.

c. The frequency distribution of lengths of insertions.

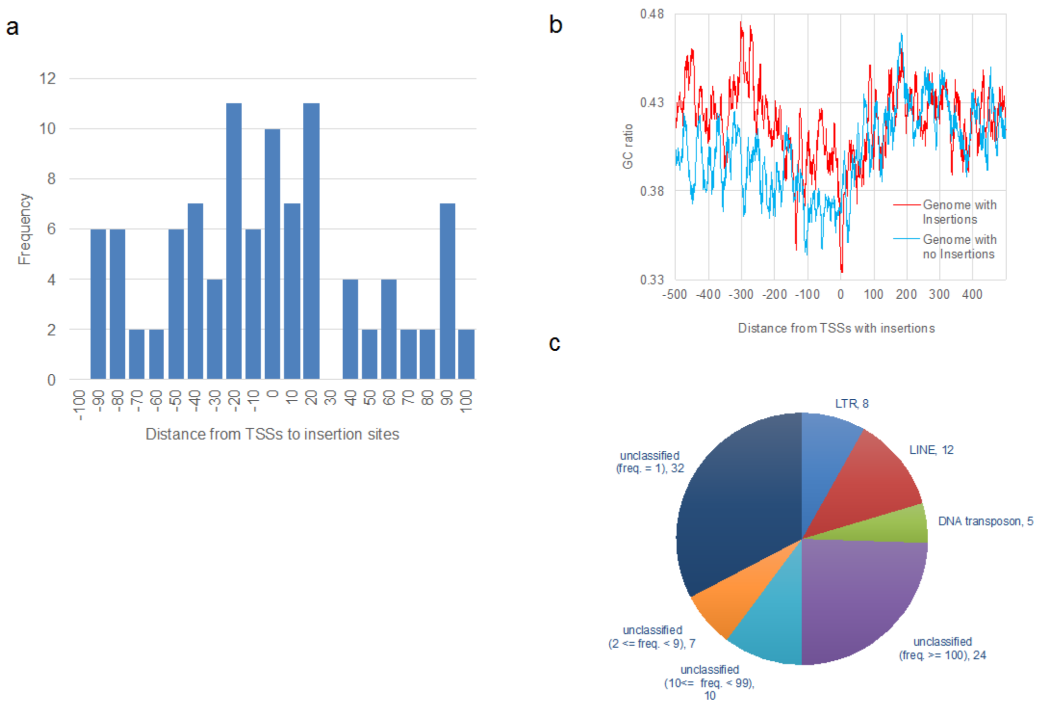

Supplementary Figure 10: Genesis of genes by mid-sized insertions into the regions upstream of transcription start sites (TSSs)

a. Frequency distribution of distances from the 101 TSSs that had increased transcript levels, to insertion sites.

b. The red and blue lines show the GC ratios around the TSSs with and without insertions, respectively.

c. Breakdown of 101 insertions according to four characterized classes (LTR, LINE, DNA transposons, and simple repeats) identified by RepeatMasker 4.0.6, and four unclassified sets grouped by their frequencies in the genomes.

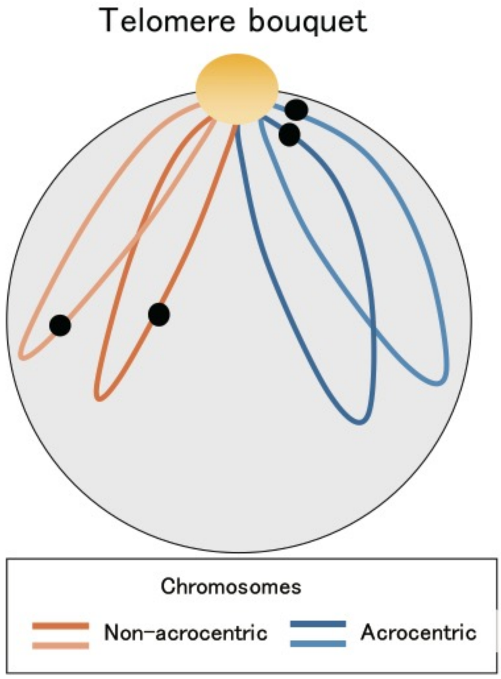

Supplementary Figure 11. Telomere bouquet and its potential relevance to the rapid evolution in non-acrocentric centromeres.

The schematic picture shows a telomere bouquet, a site on nuclear envelope where telomeres are clustered during meiotic prophase I. Centromeres of acrocentric chromosomes (blue) are brought into proximity, which may facilitate exchanges of centromeric repeats and preserve their sequence composition. By contrast, non-acrocentric centromeres (orange) are likely to be apart from each other, which may accelerate centromere evolution in individual chromosomes.

Supplementary Notes

Analysis of gaps in the assembled genomes

Despite dramatic improvements in genome assembly methods, hundreds of gaps remain in the assembled chromosomes, and we recorded ~1000 unanchored contigs. We attempted to extend contigs to the telomeric regions of individual chromosomes; however, this was successful for only a few chromosomes of each of the three strains. We also approached to the problem of how to sequence centromeres (a challenging issue in vertebrate genomics) and we found a number of contigs bearing centromeric tandem repeats could be anchored to chromosomes. At the moment, some centromeric tandem repeats still remained in unanchored contigs. We tested BioNano optical mapping technology, but the mapping data were partial, and failed to cover most of the assembled genomes. Because many contigs anchored to chromosomes were not connected by BAC-end pairs, longer reads (> 200 kbp) are required to fill the remaining gaps and allow us to identify hitherto unknown long repetitive regions.

Near-identical copies of the innate autonomous transposon *Tol*2 and the Y-specific region

To demonstrate the comprehensive nature of our current sequences, we examined the distributions of *Tol*2 element insertions. *Tol*2 is an example of an early innate autonomous transposon in a vertebrate genome [1]; *Tol*2 is 4682bp in length and is estimated to be present in ~20 near-identical copies in the medaka genome [2]. Although no full *Tol*2 matches were found in the previous Sanger-sequence genome assembly (version 1), we identified 15, 5, and 16 full matches in the new Hd-rR, HNI and HSOK genomes (version 2.2.4); BLASTn showed that all were >99.8% identical to the reference *Tol*2 sequence, except for two with identities of 99.7% and 99.4%. Supplementary Table 6 shows the locations of all *Tol*2 sequences and their levels of identity. We also observed copies of a known *Tol*2 variant with a 117-bp deletion (1793–1909bp) in an intron [3]. We found one such copy in Hd-rR and eight copies in HNI; the latter fact is remarkable because the presence of this variant was thought to occur at low frequencies [3]. In HSOK, we identified a novel variant with a 68-bp deletion (1487–1554bp) in another intron. The identity levels of individual deletion variants were quite high (> 99.7%). We confirmed, by manual inspection, that the three genomes bore *Tol*2 in different positions. This means that individual *Tol*2 copies have only recently been incorporated into each medaka lineage as a result of horizontal transfer after divergence of Hd-rR and HNI [2].

As another example of the high quality of the new Hd-rR assembly, we examined the Y-specific

region in the medaka linkage group 1 (LG1, 33.7 Mb), carrying *DMY*, the male determining gene, first non-mammalian equivalent of *SRY* [4]. When LG1 carries an insertion of a 250-kb Y-specific region, it serves as the Y chromosome, whereas LG1 without the insert serves as the X chromosome. In the new assembly, we obtained a single contig bearing the male-determining gene, *DMY*, which had mapped to three scaffolds (with gaps) in the earlier Hd-rR genome (version 1) presumably because the Y-specific region carries a number of repetitive elements proximal to *DMY* [5]. The Y-specific region of chromosome 1 is thought to be a duplicate of a region in chromosome 9[6]. Indeed, the *DMY* mRNA sequence from HNI mapped partially to chromosomes 9 of all three genomes (Supplementary Table 7).

Centromere specification is epigenetic but not defined by the underlying DNA sequences.

In non-centromeres, underlying sequence compositions were shown to largely determine hypomethylated domains (HMDs)[7] by using a support vector machine (SVM) equipped with spectrum kernel[8, 9]. SVM has been proven to be useful in predicting HMDs[7] and in listing relevant *k*-mers (strings of length *k*) that underlie in HMDs (Methods). We hypothesized that relevant *k*-mers in HMDs of non-centromeres could also characterize HMDs in centromeric repeats, but this hypothesis did not hold true (Supplementary Fig. 6a,b). We then searched for HMD-specific *k*-mers within centromeric repeats using HSOK non-acrocentric chromosomes 2, 4, and 23, which had large hypo- and hyper-methylated domains. We indeed obtained such *k*-mers (Supplementary Fig. 6c-d), but each chromosome had a unique set of relevant *k*-mers that were unlikely to occur in the other two chromosomes (Supplementary Fig. 6e,f,g). This is consistent with centromeric repeats in non-acrocentric chromosomes evolving rapidly and independently of each other, as we have seen in the analysis of centromere evolution. We confirmed the speculation that centromere specification is primarily epigenetic but not defined by the underlying DNA sequences[10].

Telomere regions

We also sought to characterize telomeres in the medaka genome contigs by using Tandem Repeats Finder (version 4.09) to detect telomeric repeats that were short tandem repeat expansions of the vertebrate telomeric sequence (TTAGGG). The Hd-rR and HNI genomes exhibited repeat telomeric repeat arrays at the ends of two chromosomes each (Supplementary Table 17; Methods). For example, a total of 1,265 telomeric repeats was found at the end of Hd-rR chromosome 23; this was the longest telomeric repeat in all contigs. Unanchored contigs of Hd-rR and HNI contained telomeric repeats were of 65,307 bp and 31,972 bp in total, suggesting that considerable numbers of

telomeric sequences remain to be anchored.

Categorization of mid-sized SVs into three categories

We categorized SVs into groups according to three mechanisms of formation, non-homologous end-joining (NHEJ) (*e.g.*, simple insertions and deletions of mobile elements); non-allelic homologous recombination (NAHR) (*e.g.*, genome duplications); and inversion (Supplementary Fig. 7a-c). The positions of NHEJ, NAHR, and inversion events in contigs are detailed in Supplementary Tables 14, 15, and 16, respectively. Supplementary Fig. 7d breaks down the numbers of each group and shows that the majority of mid-sized SVs (96.9%) was caused by NHEJ. This led us to focus on this class and determined whether each NHEJ event was an insertion into, or a deletion from, the Hd-rR and HNI genomes using the HSOK genome as an outgroup (Fig. 4a; Methods).

Figure 3c shows one illustrating example of gene conversion and NAHR. Orange and blue repeats are respectively prevalent in acrocentric and non-acrocentric chromosomes (Supplementary Fig. 5). A possible scenario for this centromere evolution is that the orange repeat jumped into the blue repeat by gene conversion to create a basic pattern, and the pattern was duplicated multiple times by unequal crossover.

Transposable elements inserted into regions upstream of TSSs

Transposable elements (TEs) could be responsible for variation in genome size and regulatory circuits[11]. We thus examined whether inserted fragments upstream of TSSs in medaka genomes were derived from TEs. Of these 101 insertions, only 25 matched LTR, LINE, or DNA transposons (Supplementary Fig. 10c; Methods). We then examined if 76 remaining insertions were repetitive by searching the genomes for their occurrences with >90% identity, and 73 were qualified. In spite of incomplete collection of repeats in RepBase, we identified 34 (46.6% of the remaining 73 insertions) as repeats with $\geq$10 occurrences.

Supplementary References

1 Koga A, S. M., Maruyama Y, Tsutsumi M, Hori H. transposable element in fish. *Nature* **383**, 30 (1996).

2 Koga, A. *et al.* Evidence for recent invasion of the medaka fish genome by the Tol2 transposable element. *Genetics* **155**, 273-281 (2000).

3 Koga, A., Sasaki, S., Naruse, K., Shimada, A. & Sakaizumi, M. Occurrence of a short variant of the Tol2 transposable element in natural populations of the medaka fish. *Genetics research* **93**,

13-21 (2011).
4 Matsuda, M. *et al.* DMY is a Y-specific DM-domain gene required for male development in the medaka fish. *Nature* **417**, 559-563 (2002).
5 Kondo, M. *et al.* Genomic organization of the sex-determining and adjacent regions of the sex chromosomes of medaka. *Genome research* **16**, 815-826 (2006).
6 Zhang, J. Evolution of DMY, a Newly Emergent Male Sex-Determination Gene of Medaka Fish. *Genetics* **166**, 1887-1895 (2004).
7 van Heeringen, S. J. *et al.* Principles of nucleation of H3K27 methylation during embryonic development. *Genome Res* **24**, 401-410 (2014).
8 Leslie, C., Eskin, E. & Noble, W. S. The spectrum kernel: a string kernel for SVM protein classification. *Pac Symp Biocomput*, 564-575 (2002).
9 Lee, D., Karchin, R. & Beer, M. A. Discriminative prediction of mammalian enhancers from DNA sequence. *Genome Res* **21**, 2167-2180 (2011).
10 McKinley, K. L. & Cheeseman, I. M. The molecular basis for centromere identity and function. *Nat Rev Mol Cell Biol* **17**, 16-29 (2016).
11 Warren, I. A. *et al.* Evolutionary impact of transposable elements on genomic diversity and lineage-specific innovation in vertebrates. *Chromosome Res* **23**, 505-531, (2015).
