# Supplementary material for: Centromere evolution and CpG methylation during vertebrate speciation
Source: Nat Commun. 2017 Nov 28;8:1833. doi: 10.1038/s41467-017-01982-7 (PMC5705604; doi:10.1038/s41467-017-01982-7)
Supplement: Supplementary file 3 — Description of Additional Supplementary Files [file 41467_2017_1982_MOESM3_ESM.pdf]

Description of Additional Supplementary Files

File Name: Supplementary Data 1
Description: Statistics of raw reads collected from the three medaka inbred strains

File Name: Supplementary Data 2
Description: Statistics of assembled contigs for the three medaka inbred strains

File Name: Supplementary Data 3
Description: Quality of three assembled genomes in terms of coverage of genes in CEGMA

File Name: Supplementary Data 4
Description: Statistics of contig lengths of the assembled genomes for the three medaka inbred strains

File Name: Supplementary Data 5
Description: Statistics of scaffold lengths of the assembled genomes for Hd-rR strain

File Name: Supplementary Data 6
Description: Alignments of the Tol2 sequence (gi|6070336|dbj|D84375.2|) to the Hd-rR/HNI/HSOK chromosomes

File Name: Supplementary Data 7
Description: Analysis of Y-specific regions in the assembled genomes for the three medaka inbred strains

File Name: Supplementary Data 8
Description: Statistics of SNP and indel rates among the assembled genomes for the three medaka inbred strains

File Name: Supplementary Data 9
Description: Statistics of RNA-seq data collected from 57 tissue types

File Name: Supplementary Data 10
Description: Supplementary Table 10: Statistics of genes predicted from RNA-seq data

File Name: Supplementary Data 11
Description: Estimated genomic abundance of centromeric repeats in SMRT subreads

File Name: Supplementary Data 12
Description: Statistics of centromeric regions identified in the chromosomes of the assembled genomes for the three medaka inbred strains

File Name: Supplementary Data 13
Description: Positions and lengths of centromeric regions identified in the chromosomes of the assembled genomes for the three medaka inbred strains

File Name: Supplementary Data 14
Description: Positions and lengths of NHEJ structural variants that were identified between Hd-rR and HNI in the assembled contigs

File Name: Supplementary Data 15
Description: Positions and lengths of NAHR structural variants that were identified between Hd-rR and HNI in the assembled contigs

File Name: Supplementary Data 16
Description: Positions and lengths of inversions that were identified between Hd-rR and HNI in the assembled contigs

File Name: Supplementary Data 17
Description: Statistics of telomere repeats detected in the assembled genomes for the three medaka inbred strains

File Name: Supplementary Data 18
Description: Numbers of bases in the assembled genomes that were corrected from Illumina short reads using Pilon

File Name: Supplementary Data 19
Description: Regions that had error rates of > 0.1%

File Name: Supplementary Data 20
Description: Positions of centromeric repeats in the regions that had error rates of > 0.1% in Supplementary Data 19

File Name: Supplementary Data 21
Description: Strain, chromosome, and number of monomers in each monomer group (cluster), and the similarities of all pairs of representative monomers
